# Spt5 histone binding activity preserves chromatin during transcription by RNA polymerase II

**DOI:** 10.15252/embj.2021109783

**Published:** 2022-02-01

**Authors:** Cecile Evrin, Albert Serra‐Cardona, Shoufu Duan, Progya P Mukherjee, Zhiguo Zhang, Karim P M Labib

**Affiliations:** ^1^ The MRC Protein Phosphorylation and Ubiquitylation Unit School of Life Sciences University of Dundee Dundee UK; ^2^ Institute for Cancer Genetics Department of Pediatrics and Department of Genetics and Development Columbia University Irving Medical Center New York NY USA

**Keywords:** chromatin, histone, RNA polymerase II, Spt5, transcription, Chromatin, Transcription & Genomics

## Abstract

Nucleosomes are disrupted transiently during eukaryotic transcription, yet the displaced histones must be retained and redeposited onto DNA, to preserve nucleosome density and associated histone modifications. Here, we show that the essential Spt5 processivity factor of RNA polymerase II (Pol II) plays a direct role in this process in budding yeast. Functional orthologues of eukaryotic Spt5 are present in archaea and bacteria, reflecting its universal role in RNA polymerase processivity. However, eukaryotic Spt5 is unique in having an acidic amino terminal tail (Spt5N) that is sandwiched between the downstream nucleosome and the upstream DNA that emerges from Pol II. We show that Spt5N contains a histone‐binding motif that is required for viability in yeast cells and prevents loss of nucleosomal histones within actively transcribed regions. These findings indicate that eukaryotic Spt5 combines two essential activities, which together couple processive transcription to the efficient capture and re‐deposition of nucleosomal histones.

## Graphical abstract

A eukaryote‐specific histone‐binding motif in a conserved PolII processivity factor is essential for yeast viability and prevents loss of nucleosomal histones from actively transcribed regions.

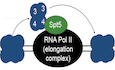

## Introduction

Eukaryotic transcription occurs in the context of chromatin, in which each transcribed nucleosome presents a barrier to RNA polymerase (Selth *et al*, 2010; Cramer, 2014; Formosa & Winston, 2020; Kujirai & Kurumizaka, 2020). The ability of RNA polymerase II (Pol II) to traverse the nucleosome barrier in eukaryotic cells is stimulated by multiple proteins, which help to weaken the association of histones with DNA and thereby facilitate Pol II progression (Schier & Taatjes, 2020). For example, the chromatin remodeller Chd1 binds to nucleosomes in a manner that displaces DNA at the Pol II entry site (Farnung *et al*, 2021). Activated by Pol II transcription into the nucleosome, Chd1 then translocates DNA into the Pol II cleft, further stimulating Pol II progression. In addition, the essential histone chaperone FACT binds to transcribing nucleosomes (Farnung *et al*, 2021) and can displace an H2A–H2B dimer, thereby facilitating Pol II progression (Belotserkovskaya *et al*, 2003; Tsunaka *et al*, 2016). Nevertheless, it is important to ensure that histones are not lost from DNA during transcription if the local pattern of nucleosome density is to be preserved. Indeed, work with budding yeast indicates that the H3–H4 tetramer from one nucleosome re‐occupies the same locus after transcription by Pol II (Henikoff & Ahmad, 2019; Schlissel & Rine, 2019). This spatial fidelity facilitates the preservation of epigenetic information in the form of post‐translational modifications of histones H3 and H4.

One contributing factor to the fate of nucleosomal histones during transcription is the rate of progression of the Pol II elongation complex. *In vitro* reactions in the absence of FACT have shown that slow transcription of a nucleosome substrate by Pol II, in the presence of limiting concentrations of nucleoside triphosphates (NTPs), allows the transfer of the histone octamer *in cis* to the upstream DNA (Bintu *et al*, 2011). Such transfer has been proposed to be mediated by loops in the DNA template (Kulaeva *et al*, 2009). However, faster Pol II elongation at more physiological NTP concentrations triggers displacement of an H2A–H2B dimer from a transcribed nucleosome (Kireeva *et al*, 2002), while even higher transcription rates can cause loss of the entire histone octamer (Bintu *et al*, 2011).

Studies of histone dynamics in living cells supported the idea that Pol II transcription frequently leads to transient loss of an H2A–H2B dimer, whereas H3–H4 tetramers are retained more stably during transcription but can be lost from very highly expressed genes, thereby reducing nucleosome density. For example, Pol II transcription was found to stimulate the exchange of histone H2A–H2B dimers within gene bodies in chicken cells (Jackson, 1990; Dion *et al*, 2007), human cells (Kimura & Cook, 2001), slime mould (Thiriet & Hayes, 2005) and budding yeast (Dion *et al*, 2007; Jamai *et al*, 2007; Yaakov *et al*, 2021). The same studies found that H3–H4 tetramers were exchanged much more slowly. However, genome‐wide studies of histone exchange in budding yeast cells showed that repeated transcription of highly expressed genes reduces nucleosome density and thereby increases the rate of exchange of histones H3–H4, within coding regions as well as at promoters (Kristjuhan & Svejstrup, 2004; Lee *et al*, 2004; Schwabish & Struhl, 2004; Jamai *et al*, 2007; Rufiange *et al*, 2007; Yaakov *et al*, 2021). Furthermore, the metazoan histone variant H3.3 is incorporated into highly transcribed genes (Mito *et al*, 2005), likely also reflecting some degree of nucleosome depletion during active transcription.

Recent structural data have indicated that the association of elongation factors with Pol II in eukaryotic cells might preclude histone transfer by a simple DNA template looping model (Ehara *et al*, 2019; Kujirai & Kurumizaka, 2020). Consistent with this view, the retention of histones H3–H4 during Pol II chromatin transcription in budding yeast was found to be an active process that involves multiple histone chaperones, including FACT and Spt6 that are essential for cell viability (Kaplan *et al*, 2003; Jamai *et al*, 2009; Perales *et al*, 2013; Jeronimo *et al*, 2019). Although FACT was first characterised as a chaperone of histone H2A–H2B, it also binds to H3–H4 tetramers *in vitro*, via a domain that is distinct from the previously characterised module that binds to H2A–H2B (Tsunaka *et al*, 2016; Mayanagi *et al*, 2019; Liu *et al*, 2020). Similarly, Spt6 binds to H3–H4 tetramers *in vitro* but is also able to associate with H2A–H2B (Bortvin & Winston, 1996; McCullough *et al*, 2015). Consistent with the essential role of FACT and Spt6 in the retention of H3–H4 tetramers during Pol II transcription in yeast cells, mutations in FACT or Spt6 lead to the potent activation of transcription within gene bodies, from cryptic promoters that are normally repressed by the presence of nucleosomes (Kaplan *et al*, 2003; Cheung *et al*, 2008).

Until now, however, it has remained unclear how progression of the Pol II elongation complex is coupled to the capture, retention and local re‐deposition of the H3–H4 tetramer by factors such as FACT and Spt6. Structural and biochemical studies have indicated that FACT is recruited to nucleosomes in a transcription dependent manner (Mayanagi *et al*, 2019; Liu *et al*, 2020), but FACT has not been found to contact the Pol II elongation complex directly (Farnung *et al*, 2021). Moreover, although Spt6 does associate with the Pol II elongation complex (Vos *et al*, 2018, 2020), mutations that displace Spt6 from Pol II were not found to impair nucleosome re‐deposition during transcription (Dronamraju *et al*, 2018). Therefore, it is currently unclear how histone chaperones such as FACT and Spt6 can promote the local retention of H3–H4 tetramers during progression through chromatin of the Pol II elongation complex.

One possibility is that the Pol II elongation complex itself plays a direct role in histone capture and redeposition during transcription, in addition to the action of chaperones such as FACT and Spt6. A clue for such a role of the Pol II elongation complex was suggested by analogous studies of histone capture and redeposition during chromosome replication. Passage of the replisome through a chromosome disassembles the downstream nucleosomes and thereby displaces parental histones, which are captured by multiple replisome components and then redeposited locally onto the newly replicated DNA (Foltman *et al*, 2013; Evrin *et al*, 2018; Serra‐Cardona & Zhang, 2018; Yu *et al*, 2018; Schlissel & Rine, 2019; Li *et al*, 2020; Stewart‐Morgan *et al*, 2020; Willhoft & Costa, 2021). The first histone‐binding component of the replisome to be identified was the MCM2 subunit of the CMG helicase (Ishimi *et al*, 1998), which unwinds the parental DNA duplex at replication forks (CMG = Cdc45‐Mcm‐GINS). The acidic amino terminal tail of MCM2 (MCM2N) binds to H3–H4 tetramers (Huang *et al*, 2015; Richet *et al*, 2015), dependent upon a conserved motif that is required to preserve repressive chromatin in yeast chromosomes (Foltman *et al*, 2013) and is important for the local redeposition of histones at replication forks in budding yeast and human cells (Gan *et al*, 2018; Petryk *et al*, 2018; Schlissel & Rine, 2019).

When extracts of budding yeast cells were treated with DNase to displace nucleosomal histones from chromatin, mass spectrometry showed that FACT forms ternary complexes *in vitro* with all four released histones, together with one of two other proteins (Foltman *et al*, 2013). The first of these was Mcm2, dependent upon the histone binding activity of Mcm2N. The second was the Spt5 subunit of the RNA Pol II elongation complex. The formation of such complexes was dependent upon the histone binding activity of FACT and the data raised the possibility that FACT forms two mutually exclusive complexes with nucleosomal histones under such conditions, one comprising Mcm2‐histones‐FACT and the second corresponding to Spt5‐histones‐FACT (Foltman *et al*, 2013). The significance of such histone‐dependent complexes containing FACT and Spt5 was not explored further in previous studies, but these findings raised the interesting possibility that Spt5 might possess an analogous histone binding activity to Mcm2.

**Figure 1:**
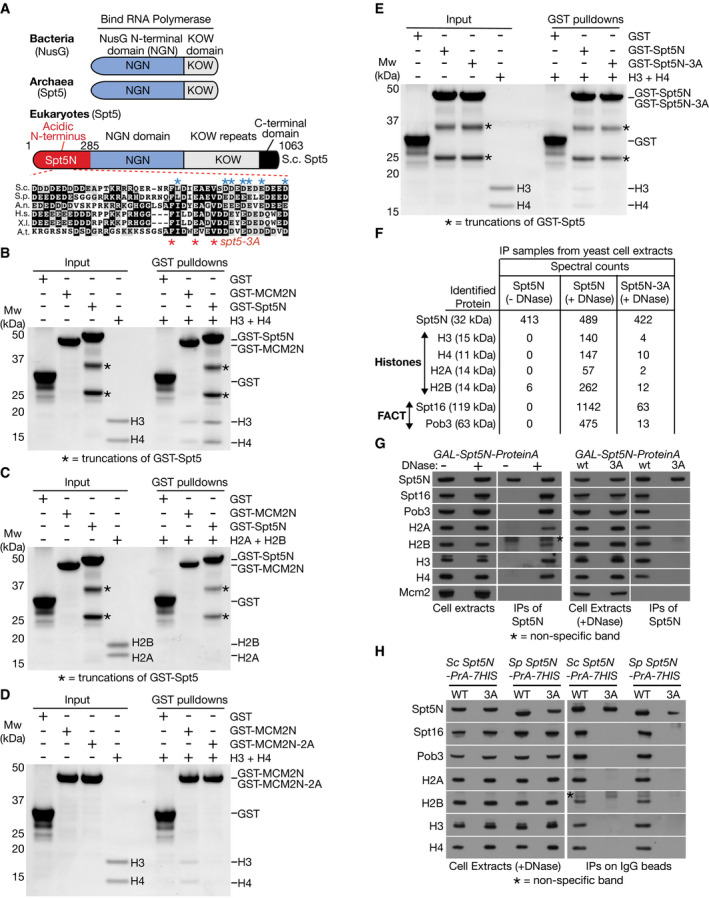
Eukaryotic Spt5N binds histones H3–H4 via an evolutionarily conserved motif Domain structure of Spt5/NusG proteins. The enlarged inset shows a conserved region of Spt5N from *Saccharomyces cerevisiae* (S.c.), *Schizosaccharomyces pombe* (S.p.), *Aspergillus nidulans* (A.n.), *Homo sapiens* (H.s.), *Xenopus laevis* (X.l.) and *Arabidopsis thaliana* (A.t.). Spt5N contains three invariant residues that were mutated in Spt5‐3A (indicated by red asterisks), whilst residues with conservative substitutions are denoted by blue asterisks.GST, GST‐MCM2N (human) and GST‐Spt5N (yeast) were mixed as indicated with yeast histones H3–H4, in the presence of glutathione agarose beads (see STAR method for further details). The input and pull‐down samples were analysed by SDS–PAGE before staining with colloidal Coomassie blue. The asterisks denote truncations of GST‐Spt5N.Similar experiment to (B) but with histones H2A–H2B.Similar experiment to (B) to compare histone binding by GST‐MCM2N and GST‐MCM2N‐2A (with Y81A Y90A mutations of conserved residues).Analogous experiment to (D), comparing histone binding by GST‐Spt5 and GST‐Spt5N‐3A (with F180A E184A V187A mutations of conserved residues).Budding yeast Spt5N and Spt5N‐3A were expressed as fusions to Protein A in budding yeast cells. Cell extracts were treated with DNase as indicated, to release nucleosomal histone complexes from chromatin, before immunoprecipitation (IP) of Spt5N/Spt5N‐3A on magnetic beads coated with IgG. The bound proteins were eluted and then analysed by mass spectrometry (the panel indicates the detected spectral counts).Samples from an analogous experiment to that shown in (F) were analysed by immunoblotting of the indicated factors. The asterisk indicates a non‐specific band in the immunoblot for histone H2B.Budding yeast cells expressing fusions of Spt5N and Spt5N‐3A to Protein A were grown in parallel with cells expressing the equivalent wild type and mutant versions of fission yeast Spt5N (Sp Spt5N and Sp Spt5N‐3A – the latter contained the F146A, E150A and V153A mutations). See also Fig EV1.

Spt5 is the only factor associated with RNA polymerase that is universally conserved in bacteria (where it is known as NusG, for N‐utilisation substance G), archaea and eukaryotes (Werner & Grohmann, 2011). The NGN domain of Spt5/NusG (NGN = NusG N‐terminal Domain) forms a stable lid over the active centre of RNA polymerase, thereby trapping nucleic acids into the cleft on the polymerase surface and ensuring processive elongation (Klein *et al*, 2011; Martinez‐Rucobo *et al*, 2011; Bernecky *et al*, 2016, 2017; Liu & Steitz, 2017). However, eukaryotic Spt5 helps RNA polymerase progress through nucleosomes (Crickard *et al*, 2017; Ehara *et al*, 2019; Farnung *et al*, 2021) and is unique in having an acidic and largely unstructured tail at the amino terminus of the protein (Spt5N), immediately before the NGN domain (Fig 1A). Recent cryoEM structures of the transcribing yeast Pol II on a nucleosome substrate indicated that Spt5N, together with the NGN domain of Spt5, is sandwiched between the downstream nucleosome and the upstream DNA that emerges from Pol II (Ehara *et al*, 2019; Farnung *et al*, 2021). Therefore, Spt5N is ideally positioned to contribute to the capture and local retention of nucleosomal histones that are released from DNA during transcription. Such a role would be consistent with previous data showing that Spt5 is important to repress cryptic transcription within gene bodies across the yeast genome (Cheung *et al*, 2008). Here, we show that Spt5N contains a conserved histone binding motif, which is essential for cell viability and preserves the integrity of nucleosomes during transcription by Pol II.

## Results and Discussion

### Eukaryotic Spt5N contains a conserved motif that binds to histones H3–H4

**Figure EV1:**
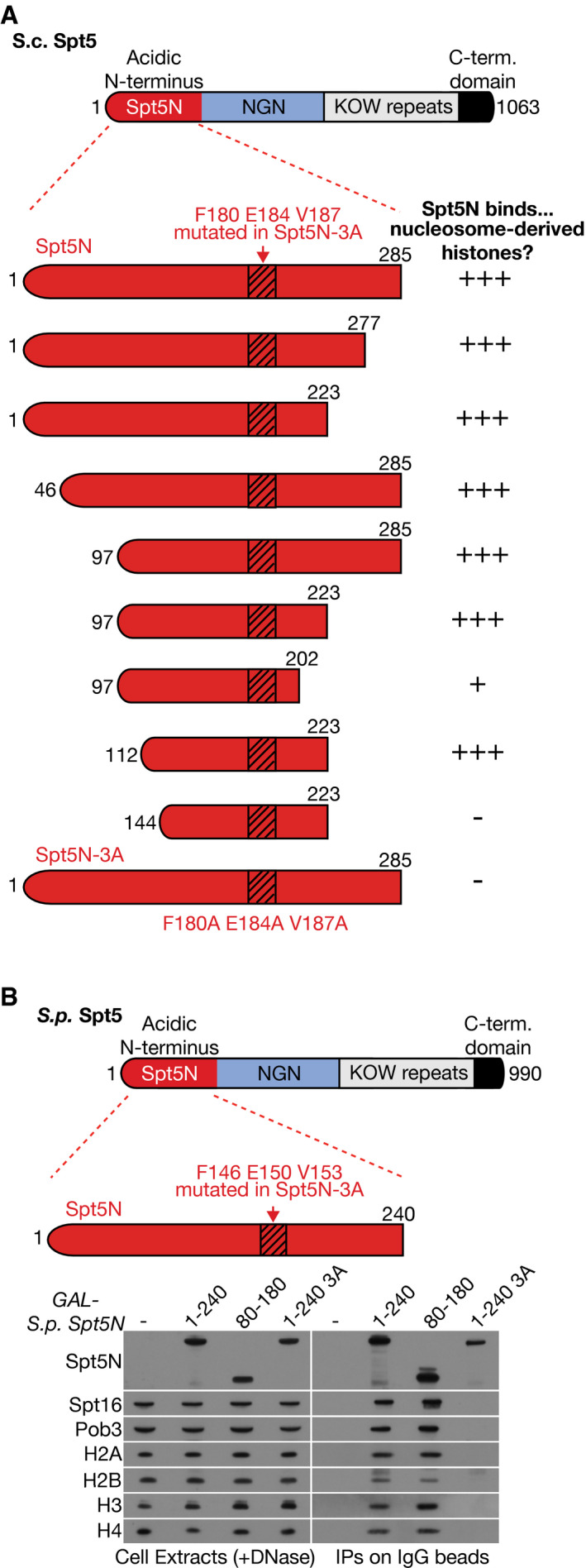
Truncation and mutation analysis of the histone binding activity of Spt5N from budding and fission yeasts Each of the indicated versions of Spt5N was expressed in budding yeast cells as a fusion to Protein A and then analysed as in Fig 1H. The association of each variant of Spt5 with nucleosome‐derived histones is summarised as shown (graded high to low as +++, + and −).In a similar experiment, the ability of the indicated variants of fission yeast Spt5N to bind nucleosome‐derived histones, together with FACT, was analysed as in Fig 1H.

To assay directly for histone binding by Spt5N via *in vitro* pull‐down experiments, we purified yeast histones and a GST‐tagged version of budding yeast Spt5N from *Escherichia coli* cell extracts, together with GST‐tagged human MCM2N as a positive control (Fig 1B and C, input). Consistent with previous reports (Huang *et al*, 2015; Richet *et al*, 2015), MCM2N bound preferentially to histones H3–H4 (Fig 1B and C, GST pulldowns), dependent upon two conserved hydrophobic residues in the histone binding motif (Fig 1D) that are essential for histone binding (Foltman *et al*, 2013; Huang *et al*, 2015). Strikingly, Spt5N also bound preferentially to histones H3–H4 (Fig 1B and C, GST pulldowns) in similar experiments. Moreover, histone binding was impaired by mutation of conserved residues in Spt5N (Fig 1E, Spt5N‐3A). To address further the specificity of these observations, we expressed a tagged version of Spt5N in budding yeast cells, incubated cell extracts in the presence or absence of nuclease to release nucleosomal histones from DNA and then purified tagged Spt5N before analysing the associated proteins by mass spectrometry. When purified from yeast cell extracts that had not been treated with nuclease, no partners of Spt5N were detected (Fig 1F and G, Spt5N −DNase). However, upon release of nucleosomal histones into the extract, Spt5N formed a specific complex with histones and FACT *in vitro*, which was largely abrogated upon mutation of the conserved residues in Spt5N that we showed above are important for histone binding (Fig 1F and G, Spt5N/Spt5N‐3A +DNase; Fig EV1A shows that histone binding was retained by a fragment containing amino acids 112–233). Taken together, these findings define a histone binding motif in Spt5N that shows preference for H3–H4. Moreover, Spt5N and FACT can bind simultaneously to histone complexes released from nucleosomes, analogous to the Mcm2N‐histone‐FACT complexes that were reported previously (Foltman *et al*, 2013).

Finally, we investigated whether the histone binding motif of budding yeast Spt5N has been functionally conserved during eukaryotic evolution. A tagged version of fission yeast Spt5N was expressed in budding yeast cells and then purified from extracts that had been treated with DNase to release nucleosomal histones from chromatin. In this way, fission yeast Spt5N was seen to form ternary complexes with histones and FACT *in vitro*, dependent upon the equivalent conserved residues to those described above for budding yeast Spt5 (Figs 1H and EV1B). These findings indicated that histone binding activity is a conserved feature of eukaryotic Spt5N.

### The histone binding activity of Spt5N is essential for yeast cell viability

**Figure 2:**
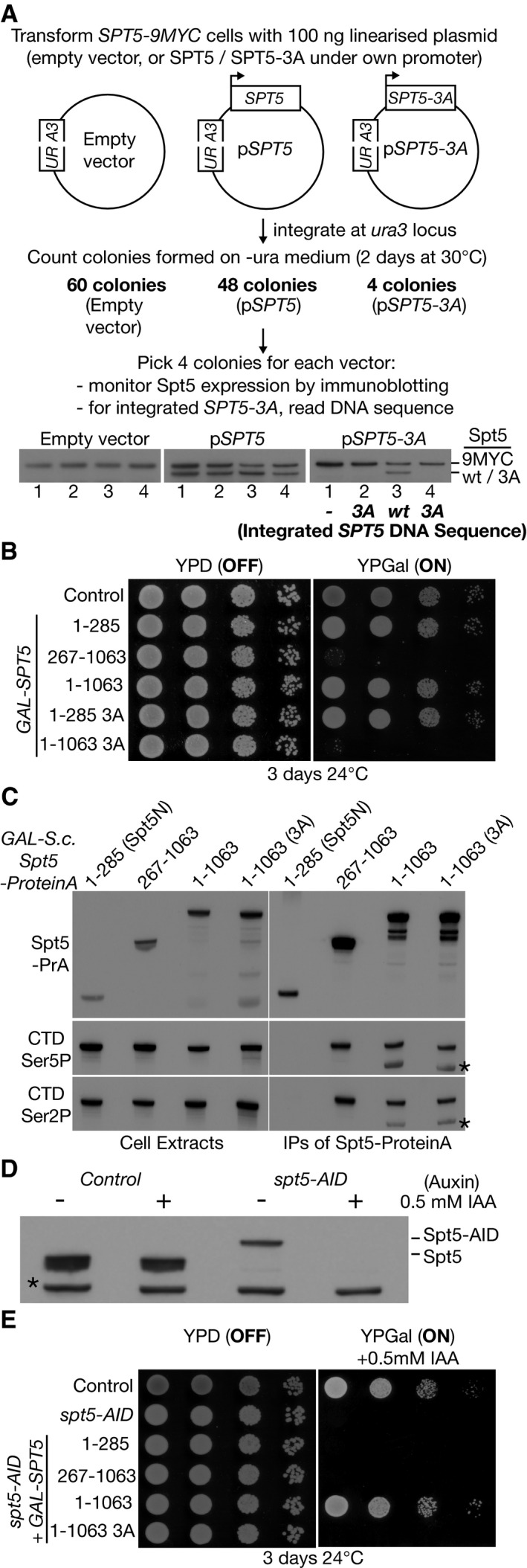
Mutation of the histone‐binding motif of Spt5N is lethal, even in the presence of wild type Spt5 Linearised versions of the indicated plasmids were transformed into *SPT5‐9MYC* budding yeast cells and integrated at the *ura3* locus. For each transformation, four colonies were analysed by immunoblotting and DNA sequencing as shown. Colonies transformed with plasmid expressing Spt5‐3A either lost the plasmid (1), contained the *SPT5‐3A* DNA but failed to express Spt5‐3A protein (2 + 4) or experienced a gene conversion and thus expressed wild type Spt5 from the integrated plasmid DNA (3).Serial dilutions of yeast cells expressing the indicated fragments of wild type Spt5 or Spt5‐3A from the *GAL1,10* promoter were grown on the indicated medium, together with a control of wild type cells.Yeast cells were generated that expressed Protein A‐tagged versions of wild type Spt5 (full length or the indicated truncations), or full length Spt5‐3A, under the control of the *GAL1,10* promoter. Cells were grown in medium containing galactose and the ProteinA‐tagged Spt5 proteins were isolated from cell extracts on magnetic beads coated with IgG. Association of the tagged Spt5 proteins with the elongating form of Pol II was monitored by immunoblotting, using antibodies specific to the indicated phosphorylation sites in the C‐Terminal Domain repeats of the Rpb1 subunit of Pol II.Control and *spt5‐AID* strains were grown at 30°C in rich medium, before addition of auxin (0.5 mM 3‐indoleacetic acid or IAA) for 1 h. Spt5 protein was monitored in cell extracts by immunoblotting.Serial dilutions of control cells or *spt5‐AID* cells, expressing the indicated versions of Spt5 from the *GAL1,10* promoter, were grown on the indicated medium.

As a first step towards exploring the functional significance of histone binding by Spt5, we tried to introduce the *spt5‐3A* mutations into one of the two copies of *SPT5* in a diploid yeast cell, with the aim of subsequently generating haploid *spt5‐3A* cells via sporulation. Intriguingly, this proved to be impossible despite repeated attempts, suggesting that mutation of the Spt5N histone‐binding motif might cause dominant lethality, when co‐expressed in cells together with wild type Spt5. We explored this possibility further in two ways. Firstly, we generated integrative vectors that targeted the yeast *ura3* locus, expressing either wild type *SPT5* or mutant *spt5‐3A* from the endogenous *SPT5* promoter, together with an empty vector control. These vectors were then transformed into cells expressing a tagged version of Spt5 from the endogenous *SPT5* locus, so that expression of untagged Spt5 from the integrated plasmids could be distinguished by immunoblotting. Transformed cells expressing a single copy of wild type Spt5 from the *ura3* locus were readily obtained (Fig 2A). In contrast, attempted integration of the plasmid containing *spt5‐3A* yielded very few colonies and subsequent analysis showed that these all lacked expression of Spt5‐3A protein (either due to a failure to express the plasmid encoded Spt5, or via a gene conversion that led to expression from the integrated plasmid of untagged wild type Spt5). Secondly, we generated haploid yeast strains expressing variants of Spt5 from the inducible *GAL1,10* promoter. Whereas expression of wild type Spt5 did not affect cell growth, induction of Spt5‐3A was lethal in wild type cells, as was expression of a truncated Spt5 protein that lacked Spt5N (Fig 2B). All three versions of Spt5 associated equivalently with Pol II (Fig 2C), whereas the isolated Spt5N domain did not bind Pol II (Fig 2C) and expression of Spt5N‐3A did not induce lethality (Fig 2B, Spt5 1–285 3A). Taken together, these findings indicated that the association of Spt5‐3A with Pol II produces a dominant lethal phenotype in wild type yeast cells, even when the wild type and mutant proteins are expressed at similar levels.

To examine the phenotype of *SPT5‐3A* in the absence of wild type Spt5, we generated yeast cells in which endogenous Spt5 was tagged with the auxin‐inducible degron (Nishimura *et al*, 2009). Spt5‐AID protein was degraded in the presence of auxin and the ubiquitin ligase OsTIR1 (Fig 2D). Correspondingly, *spt5‐aid ADH‐OsTIR1* cells grew similarly to wild type cells under permissive conditions but were inviable on medium containing auxin (Fig 2E). Importantly, the lethality of *spt5‐AID* in the presence of auxin was rescued by expression of wild type Spt5 protein, but not by expression of Spt5‐3A (Fig 2E; isolated Spt5N and Spt5 lacking Spt5N also failed to rescue *spt5‐AID*). These data indicate that the histone binding activity of Spt5N is essential for cell viability, though it is dispensable for the binding of Spt5 to Pol II.

### Mutation of the Spt5N histone‐binding motif does not block progression of the Pol II elongation complex

**Figure 3:**
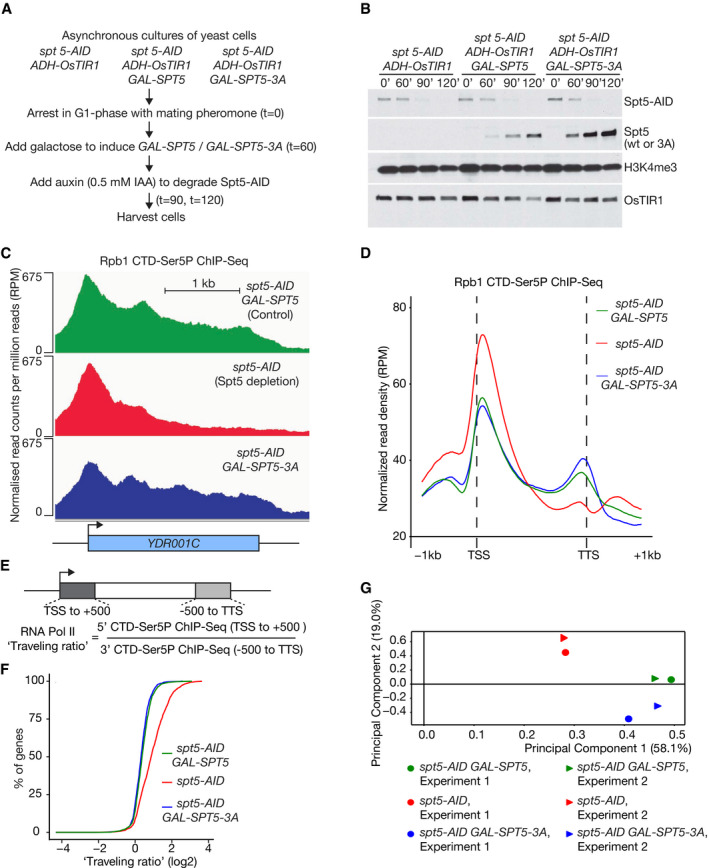
The histone binding activity of Spt5N is dispensable for progression of the Pol II elongation complex through transcription units Experimental procedure, based on the yeast strains YCE281, YCE350 and YCE356.Immunoblot analysis at the indicated times for the samples described in (A).Cells from the end of the experiment described in (A) were fixed and processed for ChIP‐Seq, using antibodies specific for phosphorylated Serine 5 in the C‐Terminal Domain of the Pol II catalytic subunit Rpb1 (CTD‐Ser5P). For each of the three indicated strains, the histograms represent the normalised read density (DNA sequence read count per million reads, or RPM), across a sample gene body.Average of the normalised read density (RPM) for the CTD‐Ser5P ChIP‐Seq data from two independent experiments, for regions spanning 1kb upstream and downstream of the transcription start site (TSS) of actively transcribed genes across the yeast genome (the analysis focussed on 821 actively transcribed genes, as described in Materials and Methods).For each transcribed gene, an RNA Pol II travelling ratio was calculated as indicated.Log_2_ values of the calculated Pol II travelling ratios for each actively transcribed gene were arranged in order from low to high and then plotted against percentile.Principal component analysis of two independent experiments, for the Pol II CTD‐Ser5P ChIP‐Seq signal across the yeast genome in the three indicated strains.

**Figure EV2:**
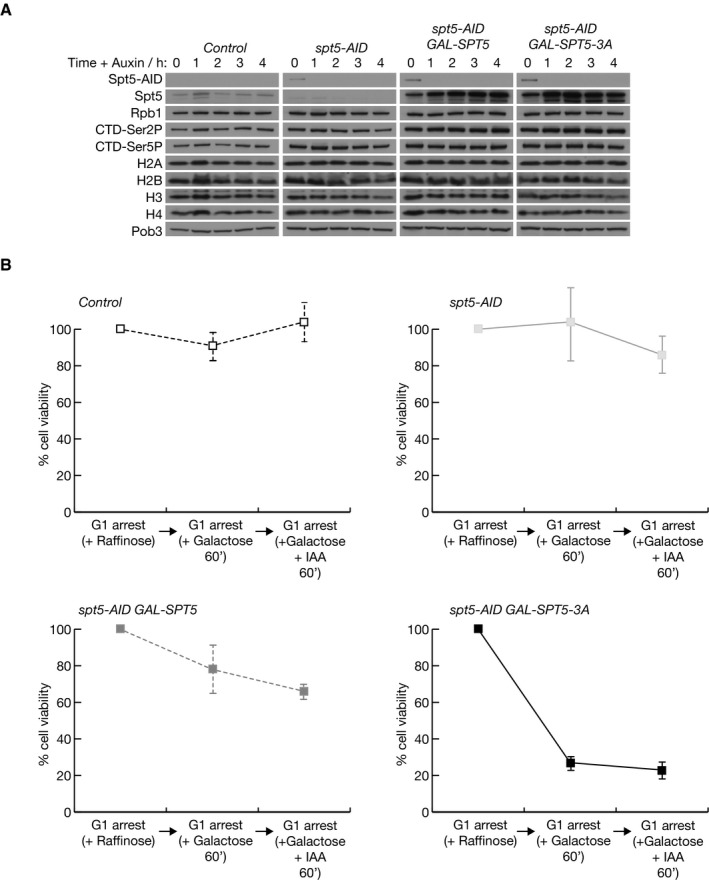
Protein levels and cell viability after transient inactivation of Spt5‐AID and expression of GAL‐SPT5 or GAL‐SPT5‐3A Cells were grown as in Fig 3A and auxin (0.5 mM IAA) was then added to the cultures for the indicated times. The indicated proteins were monitored by immunoblotting of cell extracts.At the indicated timepoints in analogous experiments to that described in Fig 3A and B, cells were sonicated and plated on rich medium lacking auxin and with glucose as the carbon source, before growth at 30°C for 2 days. Colony formation was then counted and the data represent the means and standard deviations from three biological replicates.

Since Spt5 is an essential processivity factor for Pol II during transcription, we used ChIP‐Seq to monitor the progression of the Pol II elongation complex across the budding yeast genome, in cells that either lacked Spt5, expressed wild type Spt5, or else expressed the Spt5‐3A mutant. Cells corresponding to *spt5‐AID*, *spt5‐AID GAL‐SPT5* and *spt5‐AID GAL‐SPT5‐3A* were synchronised in G1‐phase to exclude changes in gene expression during the experiment that might otherwise reflect different stages of the cell cycle. Subsequently, cells were transferred to medium containing galactose to express wild type Spt5 or Spt5‐3A, before addition of auxin for 60 min to degrade Spt5‐AID (Fig 3A and B). In contrast to recent reports (Aoi *et al*, 2021; Hu *et al*, 2021), rapid depletion of Spt5‐AID did not destabilise Rpb1 (Fig EV2A). Moreover, transient depletion of Spt5‐AID was reversible and did not lead to a permanent loss of cell viability (Fig EV2B, *spt5‐AID*), though expression of *GAL‐SPT5‐3A* reduced viability (Fig EV2B, *spt5‐AID GAL‐SPT5‐3A*), as predicted by the data described above.

**Figure EV3:**
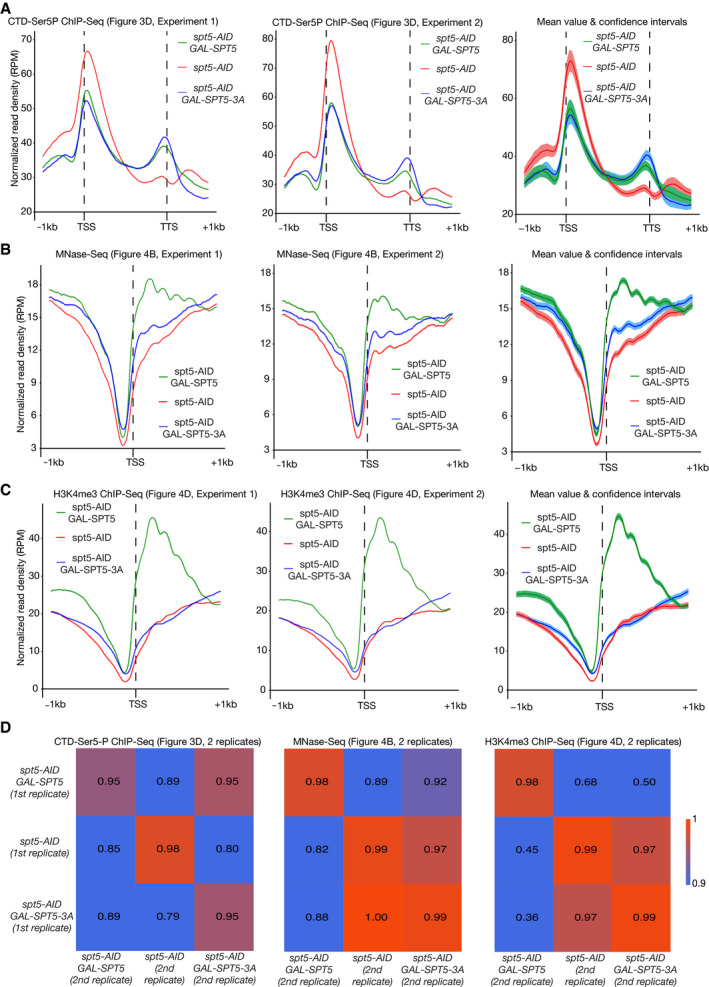
Confidence intervals and correlation coefficients for ChIP‐Seq and MNase‐Seq experiments Normalised read density (RPM) for the CTD‐Ser5P ChIP‐Seq data are shown for two biological replicates, for regions spanning 1kb upstream and downstream of the transcription start site (TSS) of actively transcribed genes across the yeast genome. The right panel shows the mean values that are depicted in Fig 3D, together with the associated confidence intervals (see Materials and Methods).Similar analysis for the MNase‐Seq data from Fig 4B.Analogous analysis for the H3K4me3 ChIP‐Seq data in Fig 4D.Pearson correlation coefficients (see Materials and Methods) of the two biological replicates for the experiments in Figs 3D and 4B and D.

Phosphorylation of Serine 5 in the C‐terminal domain (CTD) repeats of the Rpb1 RNA polymerase subunit was then monitored in ChIP‐Seq experiments, as a proxy for the Pol II elongation complex. Consistent with previous studies (Buratowski, 2009), CTD‐Ser5P was detected across transcription units in cells expressing wild type Spt5 (the analysis focused on 821 actively transcribed genes, as described in Materials and Methods), being particularly enriched in the first few hundred nucleotides at the 5′ end of genes (Figs 3C and D, and EV3A and D, *spt5‐AID GAL‐SPT5*). However, the CTD‐Ser5P signal in cells lacking Spt5 was markedly decreased in the central portion of gene bodies and towards the 3′ end of genes (Figs 3C and D, and EV3A and D, *spt5‐AID*), consistent with defective progression of the Pol II elongation complex. As an alternative way of representing these data, we compared globally the CTD‐Ser5P signal in the first 500 bp and the last 500 bp of transcription units (Fig 3E), leading to the calculation of a “travelling ratio” for Pol II (Shetty *et al*, 2017). This confirmed the decrease of Pol II towards the 3′ ends of genes across the budding yeast genome in the absence of Spt5 (Fig 3F). These data reflect the essential role of budding yeast Spt5 as a processivity factor for the Pol II elongation complex. Furthermore, the data closely resemble the defect in Pol II elongation that was previously observed in fission yeast cells upon rapid depletion of Spt5 (Shetty *et al*, 2017).

In contrast, progression of Pol II was detected across entire gene bodies in cells expressing Spt5‐3A, resembling the situation in control cells that expressed wild type Spt5 (Figs 3C and D, and EV3A and D, *spt5‐AID GAL‐SPT5*). Correspondingly, the Pol II travelling ratio for actively transcribed genes was similar across the genome of cells expressing Spt5‐3A or wild type Spt5 (Fig 3F). Furthermore, a principal component analysis of the CTD‐Ser5P datasets, which are composed of two independent repeats for each strain, indicated that the distribution of CTD‐Ser5P was similar in cells expressing wild type Spt5 or Spt5‐3A, whereas the equivalent distribution in the absence of Spt5 was distinct (Fig 3G). Therefore, these data indicated that unlike Spt5 depletion, mutation of the essential Spt5N histone‐binding motif does not block the processive transcription of Pol II at transcription units across the budding yeast genome.

### Spt5N histone binding activity preserves transcribed chromatin

**Figure 4:**
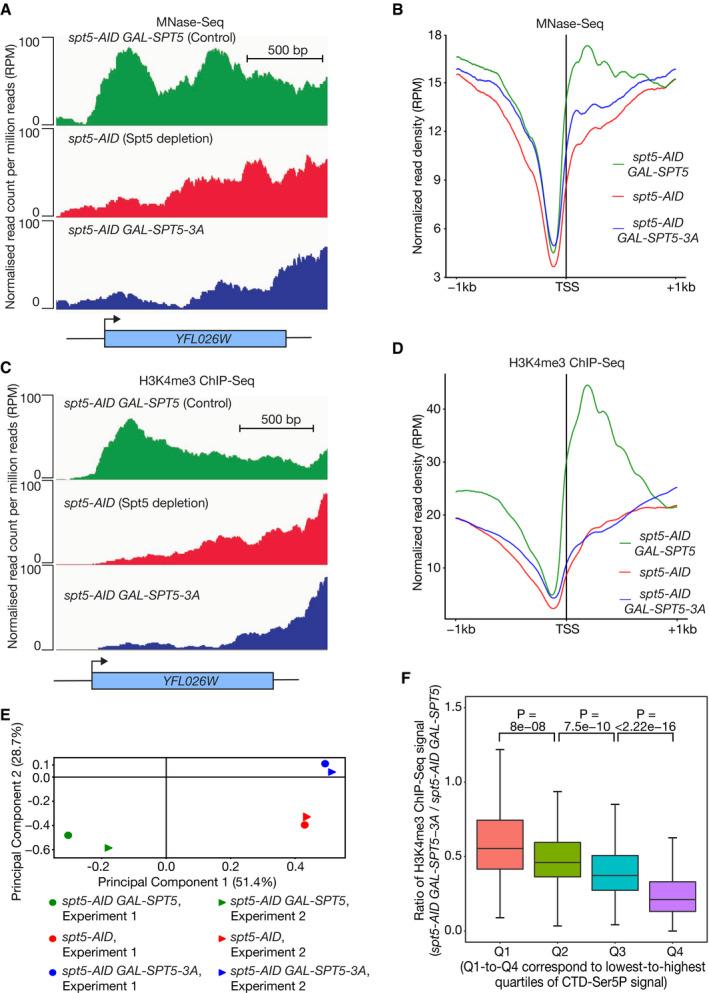
Spt5N histone binding activity preserves nucleosome integrity during transcription Samples from the experiment described in Fig 3A were also processed for MNase‐Seq. For each of the three indicated strains, the histograms represent the normalised DNA sequence read count per million reads (RPM), across a sample gene body.Average of the normalised read density (RPM) for the MNase‐Seq data from two independent experiments, for regions spanning 1kb upstream and downstream of the transcription start site (TSS) of actively transcribed genes across the yeast genome (as above, the analysis focussed on 821 active genes, as described in Materials and Methods).Samples from the same experiment were further processed for ChIP‐Seq using antibodies to histone H3K4me3. For each of the three indicated strains, the normalised read count per million reads is presented across a sample gene body.Average of the normalised read density (RPM) for the H3K4me3 ChIP‐Seq datasets from two independent experiments, for regions spanning 1kb upstream and downstream of the transcription start site (TSS) of actively transcribed genes.Principal component analysis of two independent experiments, for the H3K4me3 ChIP‐Seq signals across the yeast genome in the three indicated strains.Ratio of the H3K4me3 ChIP‐Seq signal (TSS to +500 bp) in cells expressing Spt5‐3A and cells expressing wild type Spt5. The boxes extend from the 25^th^ to 75^th^ percentiles whilst the “whiskers” illustrate the minimum and maximal values and the lines within each box correspond to the median values. The data are expressed as a function of the gene expression level, based on the read density of the ChIP‐Seq data for CTD‐Ser5P (from TSS to TTS) in Fig 3, ordered from lowest to highest values in four quartiles (colour coded as indicated). The correlation between H3K4me3 in cells expressing Spt5‐3A (*spt5‐AID GAL‐SPT5‐3A)* and control cells (*spt5‐AID GAL‐SPT5*) is progressively worse from Q1 to Q4, for genes expressed at progressively higher levels.

**Figure EV4:**
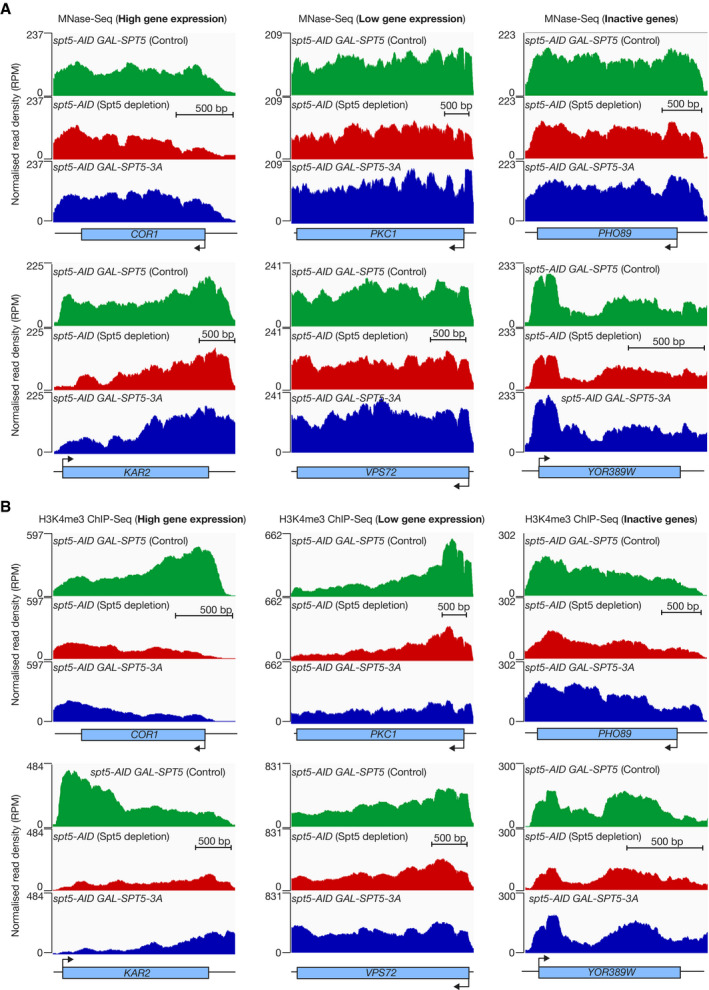
Spt5N histone binding activity is most important at highly expressed genes Further examples of MNase‐Seq data for the experiment presented in Fig 4A and B. The data correspond to two highly expressed genes (*COR1* and *KAR2*), two low expressed genes (*PKC1* and *VPS72*) and two inactive genes (*PHO89* and *YOR389W*), based on the ChIP‐Seq data for Rpb1 CTD‐Ser5P in Fig 3.Analogous data for the same six genes, from the H3K4me3 ChIP‐Seq experiment in Fig 4C and D.

Finally, we monitored the integrity of transcribed chromatin in analogous experiments to those described above, using two complementary approaches. In the first of these, we monitored nucleosome occupancy by digestion of chromatin with micrococcal nuclease (MNase) followed by high throughput DNA sequencing (MNase‐Seq). This indicated a severe drop in nucleosome occupancy within transcription units in cells that did not express wild type Spt5 (Figs 4A and B and EV3B and D; Fig EV4A illustrates that the defect was greatest for highly expressed genes). Strikingly, the reduction in nucleosome occupancy was observed both in cells lacking all forms of Spt5 (Figs 4A and B, EV3B and D, and EV4A, *spt5‐AID*) and also in cells expressing Spt5‐3A (Figs 4A and B, EV3B and D, and EV4A, *spt5‐AID GAL‐SPT5‐3A*). These findings indicated that the histone binding activity of Spt5N is important to preserve nucleosome density in transcribed regions. In the second approach, we focussed specifically on the inheritance of H3–H4 from transcribed nucleosomes, rather than studying bulk nucleosome occupancy that is also influenced by the deposition of newly synthesised histones. To do this, we used ChIP‐Seq to monitor the occupancy of nucleosomes containing trimethylation of histone H3 lysine 4 (H3K4me3). Previous studies showed that H3K4me3 is a stable mark of transcribed chromatin that persists on chromatin for several generations in yeast cells (Ng *et al*, 2003; Shilatifard, 2008; Yu *et al*, 2018; Jeronimo *et al*, 2019). Interestingly, the maintenance of H3K4me3 in transcription units was profoundly defective, both in cells lacking Spt5 and also in cells expressing Spt5‐3A (Figs 4C–E and EV3C and D). Moreover, a meta‐analysis of the data for cells expressing Spt5‐3A or wild type Spt5 indicated that the defect in the retention of H3K4me3 correlated with gene expression levels, with the greatest loss of the H3K4me3 signal in the presence of Spt5‐3A corresponding to highly expressed genes (Fig 4F; examples are provided in Fig EV4B). Therefore, these findings indicate that the histone binding activity of Spt5N is essential in budding yeast cells for the preservation of chromatin integrity during Pol II transcription.

Based on the data in this study, together with the recent observation that Spt5N and the Spt5 NGN domain are sandwiched between the downstream nucleosome and the upstream DNA during Pol II transcription (Farnung *et al*, 2021), we propose that Spt5 couples progression of the Pol II elongation complex to the local restoration of chromatin on upstream DNA, by combining two essential activities. Firstly, the NGN domain of Spt5 promotes the processivity of the Pol II elongation complex as shown previously (Bernecky *et al*, 2016, 2017; Farnung *et al*, 2021), reflecting the universal role of Spt5/NusG as a processivity factor for RNA polymerase in all living cells (Werner & Grohmann, 2011). Secondly, we propose that the newly defined histone‐binding motif in Spt5N helps to capture histones from the downstream nucleosome, to facilitate subsequent histone transfer to the adjacent upstream DNA. We show that Spt5N binds *in vitro* to histones H3–H4 and can form histone‐dependent complexes together with FACT. Such ternary complexes are likely to represent intermediates in histone transfer, which help to ensure that the H3–H4 tetramer from the downstream nucleosome is retained transiently by the Pol II elongation complex before local transfer to the upstream DNA. In future studies, it will be important to determine the structure of Spt5N in association with histones H3–H4, together with the structure of intermediates in the process of histone transfer during transcription by the Pol II elongation complex. In this context, it will be important to explore how the histone binding activities of Spt5 and FACT cooperate with Spt6 and other non‐essential histone chaperones to ensure the efficient transfer of nucleosomal histones from the downstream nucleosome to the upstream DNA. The retention of the H3–H4 tetramer during transcription is particularly important, since the tails of H3 and H4 carry post‐translational modifications that contribute to epigenetic gene regulation (Bannister & Kouzarides, 2011). However, it is possible that the H3–H4 tetramer is transferred in association with at least one H2A–H2B dimer, and we note that both FACT and Spt6 have been reported to bind all four histones under various experimental conditions (Bortvin & Winston, 1996; Belotserkovskaya *et al*, 2003; McCullough *et al*, 2015; Tsunaka *et al*, 2016; Mayanagi *et al*, 2019; Liu *et al*, 2020). Similarly, although we find that Spt5N binds preferentially to H3–H4 in our experiments, a recent structure of yeast Pol II‐Spt5‐Spt4 during transcription of a nucleosome showed that an undefined region of Spt5N can bind to the H2A–H2B dimer that is initially exposed, when transcription of a nucleosome begins and the first portion of nucleosomal DNA is displaced (Farnung *et al*, 2021). These findings suggest that Spt5, FACT and Spt6 might contact histone complexes in different ways at different stages of transcription through a nucleosome, thereby chaperoning histones upon release from downstream DNA and ensuring efficient transfer of the H3–H4 tetramer to the upstream DNA behind Pol II.

The essential role of the Spt5N histone‐binding motif in preserving nucleosomal integrity during transcription explains previous data showing that Spt5 is important to repress transcription from cryptic promoters that are located with open reading frames in the yeast genome (Cheung *et al*, 2008). Moreover, the importance of Spt5N for histone retention during transcription also explains why mutation of Spt5N causes a dominant lethal phenotype in budding yeast cells. In cells expressing both wild type Spt5 and Spt5‐3A, nucleosomal histones will be depleted from the body of actively expressed genes on each occasion that Spt5‐3A is incorporated into the Pol II elongation complex. This is likely to occur around 50% of the time in heterozygous *SPT5*/*SPT5‐3A* diploid cells, since Spt5 and Spt5‐3A are expressed to a similar level and associate equally well with Pol II. Therefore, the repeated nature of transcription means that nucleosomal histones will be rapidly depleted within highly expressed genes, in cells that express both wild type Spt5 and Spt5‐3A.

Our data highlight similarities between the role of Spt5 in the Pol II elongation complex and the role of Mcm2 in the eukaryotic replisome. Both factors are core components of their respective machineries and are essential for progression. Most importantly, both Spt5 and Mcm2 have a histone‐binding motif that is located close to the downstream nucleosome, binds H3–H4 and is important for transfer of histones to downstream DNA during transcription or replication. Moreover, both Mcm2N and Spt5N can bind simultaneously with FACT to histone complexes that have been released from nucleosomal DNA, yet such complexes containing Mcm2N or Spt5N are mutually exclusive (Fig 1F and G and (Foltman *et al*, 2013)). These features point to similarities in the mechanism of histone capture and redeposition during Pol II transcription and chromosome replication and suggest that the binding site of Spt5N on H3–H4 is likely to overlap with that of Mcm2.

Despite these similarities, the lethal phenotype of *SPT5‐3A* indicates that histone recycling at actively transcribed genes is essential for cell viability in budding yeast cells, in contrast to the recycling of parental histones during chromosome replication. This likely reflects the fact that active genes are transcribed repeatedly, whereas replication occurs just once. Therefore, defects in histone processing during transcription will lead to rapid and inescapable depletion of nucleosomes within highly active genes. Moreover, most histone synthesis is greatly stimulated during S‐phase but low during other phases of the cell cycle. This might help newly synthesised histones to compensate for a defect in parental histone recycling during replication, more easily than during transcription.

Much remains to be learnt, and we note that the ability of both the transcription and replication machineries to process nucleosomal histones requires the essential action of multiple histone chaperones: Spt5, FACT and Spt6 for Pol II transcription and Mcm2, FACT and Pol epsilon (leading strand synthesis) or Pol alpha (lagging strand synthesis) for chromosome replication. The reconstitution of such reactions with purified proteins is likely to play a central role in elucidating the underlying mechanisms in future studies, which will be important for our understanding of epigenetic inheritance in eukaryotic cells.

## Materials and Methods

Reagents, plasmids and resources from this study are listed in Table EV1.

### Purification of recombinant proteins from *E. coli* cells

#### Purification of GST and GST‐tagged Spt5N

Minimal fragments of budding yeast and fission yeast Spt5N were chosen for expression in *E. coli*, based on the analysis in Fig EV1 of histone binding in yeast cell extracts by Sc Spt5N and Sp SptN. A fragment of human MCM2N was used as a positive control for histone binding experiments and was chosen on the basis of past work (Foltman *et al*, 2013; Huang *et al*, 2015; Richet *et al*, 2015). To try and facilitate the purification of full‐length versions of the unstructured Spt5N and MCM2N variants, the chosen fragments were expressed in *E. coli* with both an amino‐terminal GST tag (GST = Glutathione *S*‐Transferase) and also a carboxy‐terminal tag that either comprised 8His or Streptag. As a negative control for histone‐binding experiments, we also expressed and purified a GST‐6His protein. The various plasmids are described in Table EV1.

Each plasmid was transformed into BL21 (DE3) Rosetta pLysS *E. coli* cells and a 1 l culture was grown in LB medium + 30 μg/ml chloramphenicol + 30 μg/ml kanamycin at 37°C. When the OD600nm reached 0.6–0.7, protein expression was induced by addition of 0.4 mM Isopropyl‐β‐d‐Thiogalactopyranoside (IPTG) for 3 h. Cells were centrifuged at 3,483 *g* for 10 min and the pellet resuspended in 25 ml lysis buffer (50 mM Tris–HCl pH8.0, 10% glycerol, 0.1% NP‐40, 10 mM MgCl_2_, 150 mM NaCl, 5 mM β‐mercaptoethanol), supplemented with 1X Halt Protease Inhibitor cocktail (PN78439 Thermo Fisher Scientific). Cells were lysed by addition of 1 mg/ml lysozyme followed by sonication for 2 min (5 s on, 5 s off) at 8% amplitude in a Soniprep 150 plus (MSE). After incubation for 30 min with Universal nuclease (123991963, Thermo Fisher Scientific; used at 100 units/g cell pellet), the lysate was centrifuged at 27,000 *g* for 20 min at 4°C. The supernatant was then mixed with 4 ml 50% slurry of Glutathione Sepharose 4B resin (11‐0756‐01, Thermo Fisher Scientific) and incubated at 4°C for 2 h. After washing with 30 column volumes of lysis buffer and 20 column volumes of 50 mM Tris–HCl pH 8.0, 50 mM KCl, 20 mM MgCl_2_, 5 mM ATP to remove contaminating heat shock proteins, the GST‐tagged protein was eluted with 6 column volumes of elution buffer (lysis buffer + 20 mM Glutathione). The fractions containing the protein of interest were identified, pooled and then mixed with 2 ml 50% slurry of either StrepTactin Superflow resin (2‐1206‐025, IBA GmbH) or Ni‐NTA agarose resin (30210, Qiagen), depending on the identity of the C‐terminal tag, before further incubation for 90 min at 4°C.

For GST‐tagged proteins with a C‐terminal 8His‐tag, the Ni‐NTA resin was then washed with 30 column volumes of “HIS‐wash buffer” (50 mM Tris–HCl pH 8.0, 0.02% NP‐40, 150 mM NaCl, 20 mM imidazole, 5 mM MgCl_2_, 5 mM β‐mercaptoethanol). The HIS‐tagged proteins were eluted with 6 column volumes of “HIS‐elution buffer” (50 mM Tris–HCl pH 8.0, 0.02% NP‐40, 150 mM NaCl, 250 mM imidazole, 5 mM MgCl_2_, 5 mM β‐mercaptoethanol).

For GST‐tagged proteins with a C‐terminal Streptag, the StrepTactin resin was washed with 30 column volumes of “Strep‐wash buffer” (50 mM Tris–HCl pH 8.0, 0.02% NP‐40, 150 mM NaCl, 5 mM MgCl_2_, 5mM β‐mercaptoethanol). The bound proteins were then eluted with 6 column volumes of “Strep‐elution buffer” (50 mM Tris–HCl pH 8.0, 0.02% NP‐40, 150 mM NaCl, 7 mM desthiobiotin, 5 mM MgCl_2_, 5 mM β‐mercaptoethanol).

Peak fractions containing the purified protein of interest were pooled and dialysed overnight at 4°C against 50 mM Tris–HCl pH 7.5, 10% glycerol and 150 mM NaCl, before being snap‐frozen in liquid nitrogen and stored at −80°C.

#### Purification of yeast histones

Untagged budding yeast histones were expressed and purified as described previously (Kingston *et al*, 2011), using plasmids shown in Table EV1. Plasmids expressing either H2A–H2B or H3–H4 were transformed into BL21 (DE3) Rosetta pLysS *E. coli* cells, before growing 1 litre cultures at 37°C in LB medium containing 30 μg/ml chloramphenicol and either 30 μg/ml spectinomycin (H2A–H2B) or 30 μg/ml ampicillin (H3–H4). When the OD600 nm reached 0.5–0.7, protein expression was induced by addition of 0.5 mM IPTG for 4 h. Cells were centrifuged at 3,483 *g* for 10 min and the pellet was resuspended in lysis buffer (20 mM Tris–HCl pH 8.0, 500 mM NaCl, 5 mM EDTA, 10 mM β‐mercaptoethanol and 1X “Complete protease inhibitor Cocktail” (11873580001, Roche; a 25× stock was made by dissolving one tablet in 1ml of water). Cells were lysed by sonication for 2 min (5 s on, 5 s off) at 8% amplitude in a MSE Soniprep 150 plus. After incubation for 30 min with 100 units of Universal nuclease per gram of cells, the lysate was centrifuged at 27,000 *g* for 30 min at 4°C. The supernatant was loaded onto a 5 ml Heparin HiTrap column (GE Healthcare) pre‐equilibrated in buffer A (20 mM Tris–HCl pH 8.0, 5 mM EDTA, 10 mM β‐mercaptoethanol). The column was then washed with 5 column volumes of buffer A and the bound proteins were eluted by a gradient over 15 column volumes from 0–100% buffer B (20 mM Tris–HCl pH 8.0, 2 M NaCl, 5 mM EDTA, 10 mM β‐mercaptoethanol). Peak fractions containing either H2A–H2B or H3–H4 were pooled, concentrated with a 10 kDa Amicon Ultra Centrifugal filter (Millipore) and then loaded onto a Superdex 200 16/600 gel filtration column, pre‐equilibrated in “Gel filtration buffer” (20 mM Tris–HCl pH 8.0, 2 M NaCl, 0.1 mM EDTA, 10 mM β‐mercaptoethanol). The fractions containing either H2A–H2B dimer or H3–H4 tetramer were pooled and concentrated before being snap‐frozen in liquid nitrogen and stored at −80°C.

### GST pull‐down assays with purified Spt5N/MCM2N and yeast histones

To monitor histone binding to Spt5N and MCM2N, 4 μg of the corresponding GST‐tagged proteins were incubated with 30 μl 50% slurry of Glutathione sepharose 4B resin in buffer C (50 mM Tris–HCl pH 7.5, 0.25% NP‐40) supplemented with 150 mM NaCl, for 30 min at 4°C. Following two washes with buffer C containing 300 mM NaCl, 0.2 μg of either H2A–H2B or H3–H4 were added to the bead suspension in buffer C containing 300 mM NaCl, before incubation for 30 min at 4°C. The beads were then washed six times for 5 min with Buffer C containing 300 mM NaCl (Fig 1B–D) or twice with Buffer C plus 500 mM NaCl (Fig 1E), before boiling in 1X Laemmli buffer. The input and eluted samples were analysed on NuPage Novex 4–12% Bis‐Tris gels (WG1402BOX, Thermo Fisher Scientific) in 1X NuPage MES SDS running buffer (NP0002, Thermo Fisher Scientific), before detection by staining with Coomassie blue.

### Yeast strains and growth

All *Saccharomyces cerevisiae* strains used in this study are listed in Table EV1. Cells were grown at 30°C in rich medium (1% yeast extract, 2% peptone) supplemented with 2% Raffinose (YPRaff), 2% Galactose (YPGal) or 2% Glucose (YPD). Cells were arrested in the G1‐phase or G2‐M phase of the cell cycle by 2‐h treatment of an exponentially growing culture (at 7 × 10^6^ cells/ml) respectively with 5 µg/ml alpha factor mating pheromone (synthesised by EZBiolab) or 5 μg/ml nocodazole (M1404, Sigma‐Aldrich). Degradation of Spt5‐AID was induced by incubating cells for the indicated times with 0.5 mM 3‐indoleacetic acid or IAA (I3750‐5G‐A, Sigma‐Aldrich).

### Serial dilution of yeast cells to monitor growth and viability on different media

For the experiments in Fig 2B and E, cells from a fresh YPD plate were diluted to 3 × 10^6^, 3 × 10^5^, 3 × 10^4^ and 3 × 10^3^ cells/ml in phosphate buffered saline (PBS). Subsequently, aliquots containing 50 × 10^4^, 50 × 10^3^, 50 × 10^2^ or 50 cells were placed in a spot on the indicated medium in a 9‐cm petri dish (YPD, YPGal or YPGal + 0.5 mM Auxin), before growth for 2 days at 30°C or 3 days at 24°C. The plates were then scanned to record images of the yeast cell colonies.

For the experiment in Fig EV2B, cell cultures were sonicated and diluted in PBS, before 500 cells were plated on YPD medium. Colonies were allowed to grow for 2 days at 30°C before counting, and the data represent the mean and standard deviations from three biological replicates.

### Monitoring the *in vitro* association of Spt5N with histones and FACT in yeast cell extracts

Budding yeast strains expressing variants of Spt5N from the GAL1,10 promoter are listed in Table EV1. One litre cultures were grown in YPRaff medium at 30°C and arrested in G2‐M phase as described above, to avoid any complications due to differing cell cycle stages. Subsequently, expression from the GAL1,10 promoter was induced by transferring cells to fresh YPGal medium containing nocodazole for 2 h. Frozen yeast pellets were prepared as described previously (Maric *et al*, 2014) in 100 mM Hepes‐KOH pH 7.9, 100 mM KOAc, 10 mM Mg(OAc)_2_, 2 mM EDTA, 2 mM β‐glycerophosphate, 2 mM NaF, 1 mM DTT, 1% Protease inhibitor Cocktail (P8215, Sigma‐Aldrich), 1× “Complete protease inhibitor Cocktail” (11873580001, Roche; a 25× stock was made by dissolving one tablet in 1ml of water). For each sample, 2–3 g of frozen cells were ground in a SPEX SamplePrep 6780 Freezer/Mill with two cycles of 2 min at a rate of 14 cycles per second. To release nucleosomal histones into the extract, the chromosomal DNA was then digested with “Universal nuclease” (123991963, Thermo Fisher Scientific; 1,600 units/ml) for 30 min at 4°C, before two consecutive centrifugations at 25,000 *g* for 30 min and 100,000 *g* for 1 h. The resulting cell extract was added to magnetic beads (Dynabeads M‐270 Epoxy, 14302D, Life Technologies) coupled to anti‐sheet IgG (S1265, Sigma‐Aldrich), before incubation for 2 h at 4°C. Bound proteins were separated on NuPage Novex 4–12% Bis‐Tris gels (WG1402BOX, Thermo Fisher Scientific) in 1X NuPage MES SDS running buffer (NP0002, Thermo Fisher Scientific), and detected by either by immunoblotting (see below) or by mass spectrometry analysis. In the latter case, gels were stained with colloidal coomassie (Simply Blue, LC6060, Thermo Fisher) and each sample lane was then cut into 40 bands before digestion with trypsin. Peptides were analysed by nano liquid chromatography tandem mass spectrometry with an Orbitrap Velos (MS Bioworks).

### Monitoring the association of Spt5N with Pol II in yeast cell extracts

For the experiment in Fig 2C, budding yeast strains expressing the indicated variants of Spt5N from the GAL1,10 promoter (listed in Table EV1) were grown and processed as above. The phosphorylated forms of the Rpb1 subunit of Pol II (CTD‐Ser5P and CTD‐Ser2P) were detected using the antibodies shown in Table EV1.

### Making denatured yeast protein extracts using trichloroacetic acid

For the experiments in Figs 2D and EV2A, ~10^8^ cells were used to prepare total protein extracts in the presence of trichloroacetic acid as described previously (Foiani *et al*, 1994).

### Immunoblotting

The primary and secondary antibodies used for immunoblotting, together with the corresponding dilutions and other details, are given in Table EV1.

### ChIP‐Seq to monitor progression of the Pol II elongation complex across the yeast genome

For the experiment in Figs 3 and EV3A, 50 ml cultures of exponentially growing yeast cells in YPRaff were arrested in G1 phase at 30°C as described above (two biological replicates), to avoid any complications due to differing cell cycle stages. Expression of *GAL‐SPT5* and *GAL‐SPT5‐3A* was then induced by transferring cells to YPGal medium for 60 min. Subsequently, Spt5‐AID was degraded by addition of IAA. For ChIP‐Seq, cells were cross‐linked with 1% paraformaldehyde for 20 min at room temperature, followed by the addition of 0.125 M glycine for 5 min at room temperature. Cells were then collected by centrifugation and resuspended in “ChIP lysis buffer” (50 mM HEPES/KOH pH 7.5, 140 mM NaCl, 1 mM EDTA, 1% Triton X‐100, 0.1% Na‐deoxycholate) containing 1× SIGMAFAST Protease Inhibitor cocktail (S8830, Sigma‐Aldrich). Cells were lysed with glass beads and bead beating 4 times for 30 s in a Mini‐Beadbeater‐16 (607, BioSpec) and chromatin was then pelleted, washed with the above buffer, and sheared by sonication using a Bioruptor Pico (Diagenode, 15″ ON, 30″ OFF, 10 cycles) to an average fragment size of 200–400 bp. Samples were centrifuged at 10,000 *g* for 10 min at 4°C and the supernatant was used for immunoprecipitation with an antibody to Rpb1 CTD‐Ser5P (04‐1572, Millipore) and protein G Sepharose beads (17‐0618‐02, GE Healthcare). DNA was recovered from both input and ChIP samples via the Chelex‐100 protocol ((Nelson *et al*, 2006), 142‐1253, Biorad) and purified with MinElute PCR kit (28004, QIAGEN). Sequencing libraries were then prepared with the Accel‐NGS 1S Plus DNA library kit (10096, Swift BioSciences).

### MNase‐Seq and H3K4me3 ChIP‐Seq to monitor nucleosome occupancy and H3K4me3 across the yeast genome

For the experiments in Figs 4, EV3 and EV4, yeast cells were grown, collected, and chromatin was prepared as described above (two biological replicates). Chromatin was washed twice with NP buffer (1.6 M sorbitol, 2 mM CaCl_2_, 5 mM MgCl, 50 mM NaCl, 14 mM β‐mercaptoethanol, 10 mM Tris–HCl [pH 7.4], 0.075% NP‐40, 5 mM spermidine) and then resuspended in the same buffer. Chromatin digestion was performed with the addition of 5 units of MNase (9013‐53‐0, Worthington Biochemical) at 37°C for 20 min, yielding a majority of mono‐ and di‐nucleosome fragments, followed by the addition of 7 mM EDTA and 1/5 volume of 5X ChIP lysis buffer (see above) on ice for 10 min. Samples were centrifuged at 10,000 *g* for 10 min at 4°C and the supernatant was used for immunoprecipitation with anti‐H3K4me3 antibody (ab8580, Abcam) and protein G Sepharose beads (17‐0618‐02, GE Healthcare). DNA was recovered as described above, from both the input (MNase‐Seq) and H3K4me3 ChIP‐Seq samples, which were then purified with MinElute PCR kit (QIAGEN) before preparing sequencing libraries.

### Data analysis of MNase‐Seq and ChIP‐Seq datasets

The MNase‐Seq and ChIP‐Seq libraries were sequenced using the paired‐end method with Illumina sequencing platforms (Hi‐seq 2000 and 2500). Raw reads were trimmed to remove sequencing adaptors and low‐quality reads, using Trim Galore (version 0.6.7) with default parameters. Filtered reads were first aligned to the *S. cerevisiae* reference genome (UCSC sacCer3) using version 2.2.4 of the short read aligner Bowtie 2 (Langmead & Salzberg, 2012). Only paired‐end reads with both ends mapped correctly were selected for subsequent analysis, after removing multi‐mapped reads via version 1.11 of SAMtools (Danecek *et al*, 2021) together with reads generated by PCR duplication via version 2.23.8 of Picard (http://broadinstitute.github.io/picard/). Coverage for each base pair of the *S. cerevisiae* genome was computed using version 2.29.2 of “genomeCoverageBed” from BEDTools (Quinlan & Hall, 2010) and then normalised to library size (reads‐per‐million or RPM). “Bedgraph” files were converted to “bigwig” files for data visualisation using version 359 of UCSC bedGraphToBigWig (Kent *et al*, 2010). Transcription start sites (TSS) and transcription termination sites (TTS) were annotated from the Saccharomyces Genome Database or SGD (Nagalakshmi *et al*, 2008). A list of 821 transcriptionally active genes (Table EV2) was identified with the following parameters: genes with a minimum length of 1,000 bp, a “Fragment Per Kilobase Million” or FPKM value of > 5 according to RNA‐Seq data of G1‐arrested cells (Feng *et al*, 2016), and belonging to the top 30% in our Rpb1 CTD‐Ser5P ChIP‐Seq dataset, which reflects elongating RNA Pol II. For the MNase‐Seq, Rpb1 CTD‐Ser5P ChIP and H3K4me3 ChIP datasets, the enrichment around the TSS of actively transcribed genes was calculated using computeMatrix from version 3.2.1 of deepTools (Ramirez *et al*, 2016). All replicates showed a Pearson correlation coefficient of greater than 0.95 for transcriptionally active genes. The travelling ratio for RNA Pol II in actively transcribed genes was calculated as described previously (Shetty *et al*, 2017). Briefly, Rpb1 CTD‐Ser5P ChIP signals were calculated 500 bp downstream from the TSS (5′ region signal) and 500 bp upstream from the TTS (3′ region signal) for each actively transcribed gene. The travelling ratio was defined as the ratio between the 5′ region signal and the 3′ region signal. PCA analysis based on the density of Rpb1 CTD‐Ser5P or H3K4me3 across the whole yeast genome was calculated using multiBigwigSummary from deepTools.

To calculate confidence intervals for the data in Fig EV2, we firstly combined the density matrix of active genes (Table EV1) from two repeats at each position. We then calculated the average, upper limit and lower limit of the 95% confidence interval using a confidence interval function at each position.

## Author contributions

**Cecile Evrin:** Conceptualization; Data curation; Formal analysis; Validation; Investigation; Visualization; Methodology; Writing—review and editing. **Albert Serra‐Cardona:** Data curation; Formal analysis; Validation; Investigation; Visualization; Methodology; Writing—review and editing. **Shoufu Duan:** Data curation; Formal analysis; Validation; Investigation; Visualization; Methodology; Writing—review and editing. **Progya, M Mukherjee:** Investigation. **Zhiguo Zhang:** Formal analysis; Supervision; Funding acquisition; Methodology; Project administration; Writing—review and editing. **Karim Labib:** Conceptualization; Formal analysis; Supervision; Funding acquisition; Visualization; Methodology; Writing—original draft; Project administration.

In addition to the CRediT author contributions listed above, the contributions in detail are:

The experiments in Figs 1, 2, EV1 and EV2 were performed by CE, whereas the experiments in Figs 3, 4, EV3 and EV4 were performed by ASC with data analysis by SD and ZZ. PPM generated plasmids pCE169‐170 and yeast strains YCE1140‐1141. The project was designed by CE and KL. KL wrote the manuscript with critical input from the other authors.

## Supporting information



Expanded View Figures PDF

Table EV1

Table EV2

## Data Availability

All raw and analysed sequencing data have been deposited in the NCBI under the GEO accession number GSE181901 (https://www.ncbi.nlm.nih.gov/geo/query/acc.cgi?acc=GSE181901).
