# Supplementary material for: Spt5 histone binding activity preserves chromatin during transcription by RNA polymerase II
Source: EMBO J. 2022 Feb 1;41(5):e109783. doi: 10.15252/embj.2021109783 (PMC8886531; doi:10.15252/embj.2021109783)
Supplement: Supplementary file 1 — Expanded View Figures PDF [file EMBJ-41-e109783-s003.pdf]

Expanded View Figures

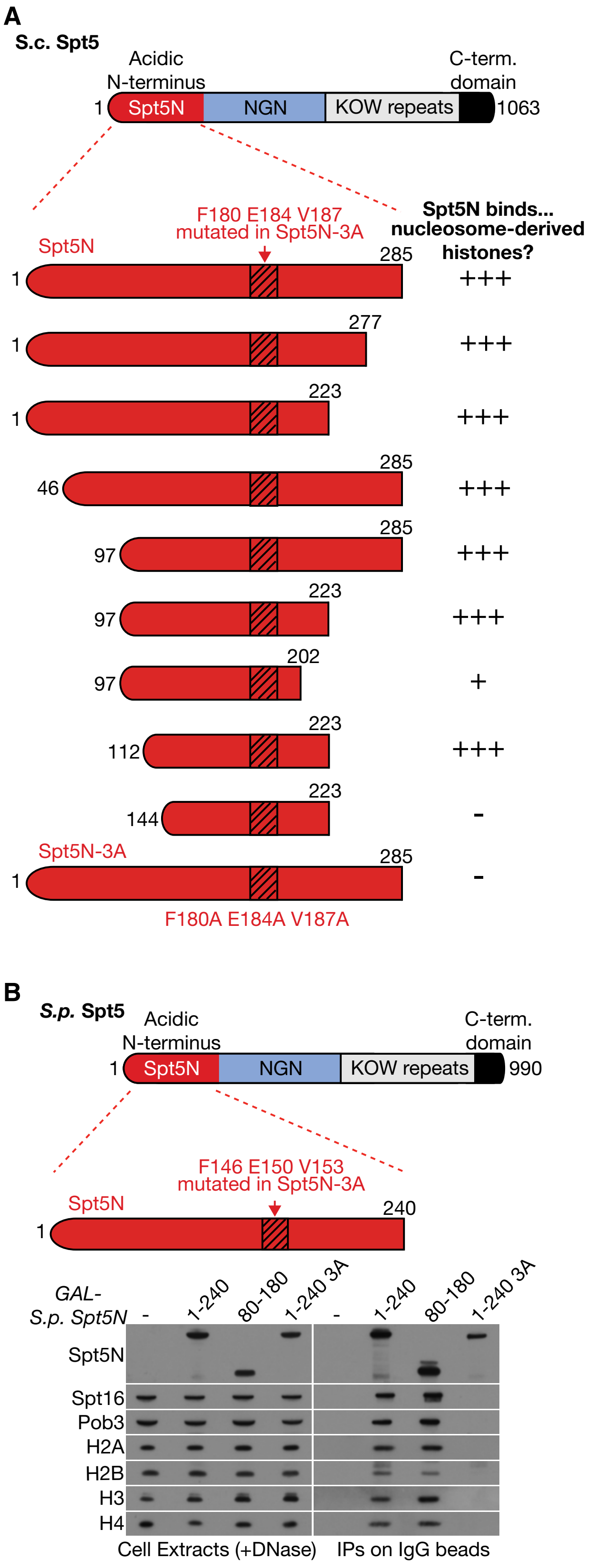

Figure EV1. Truncation and mutation analysis of the histone binding activity of Spt5N from budding and fission yeasts.

A Each of the indicated versions of Spt5N was expressed in budding yeast cells as a fusion to Protein A and then analysed as in Fig 1H. The association of each variant of Spt5 with nucleosome-derived histones is summarised as shown (graded high to low as +++, + and –).

B In a similar experiment, the ability of the indicated variants of fission yeast Spt5N to bind nucleosome-derived histones, together with FACT, was analysed as in Fig 1H.

© 2022 The Authors

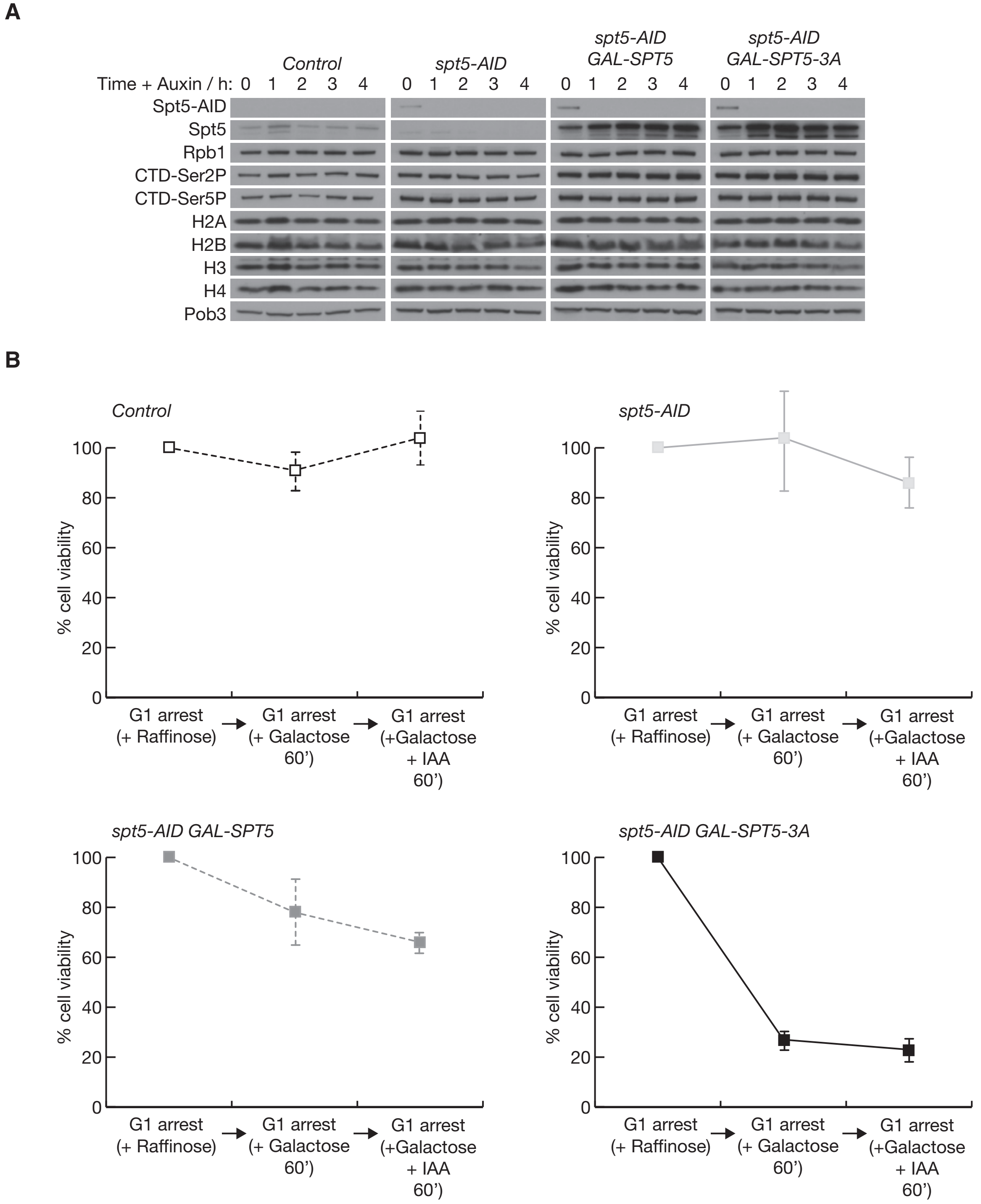

Figure EV2. Protein levels and cell viability after transient inactivation of Spt5-AID and expression of GAL-SPT5 or GAL-SPT5-3A.

A Cells were grown as in Fig 3A and auxin (0.5 mM IAA) was then added to the cultures for the indicated times. The indicated proteins were monitored by immunoblotting of cell extracts.

B At the indicated timepoints in analogous experiments to that described in Fig 3A and B, cells were sonicated and plated on rich medium lacking auxin and with glucose as the carbon source, before growth at 30°C for 2 days. Colony formation was then counted and the data represent the means and standard deviations from three biological replicates.

© 2022 The Authors

Figure EV3. Confidence intervals and correlation coefficients for ChIP-Seq and MNase-Seq experiments.

A Normalised read density (RPM) for the CTD-Ser5P ChIP-Seq data are shown for two biological replicates, for regions spanning 1kb upstream and downstream of the transcription start site (TSS) of actively transcribed genes across the yeast genome. The right panel shows the mean values that are depicted in Fig 3D, together with the associated confidence intervals (see Materials and Methods).
B Similar analysis for the MNase-Seq data from Fig 4B.
C Analogous analysis for the H3K4me3 ChIP-Seq data in Fig 4D.
D Pearson correlation coefficients (see Materials and Methods) of the two biological replicates for the experiments in Figs 3D and 4B and D.

© 2022 The Authors

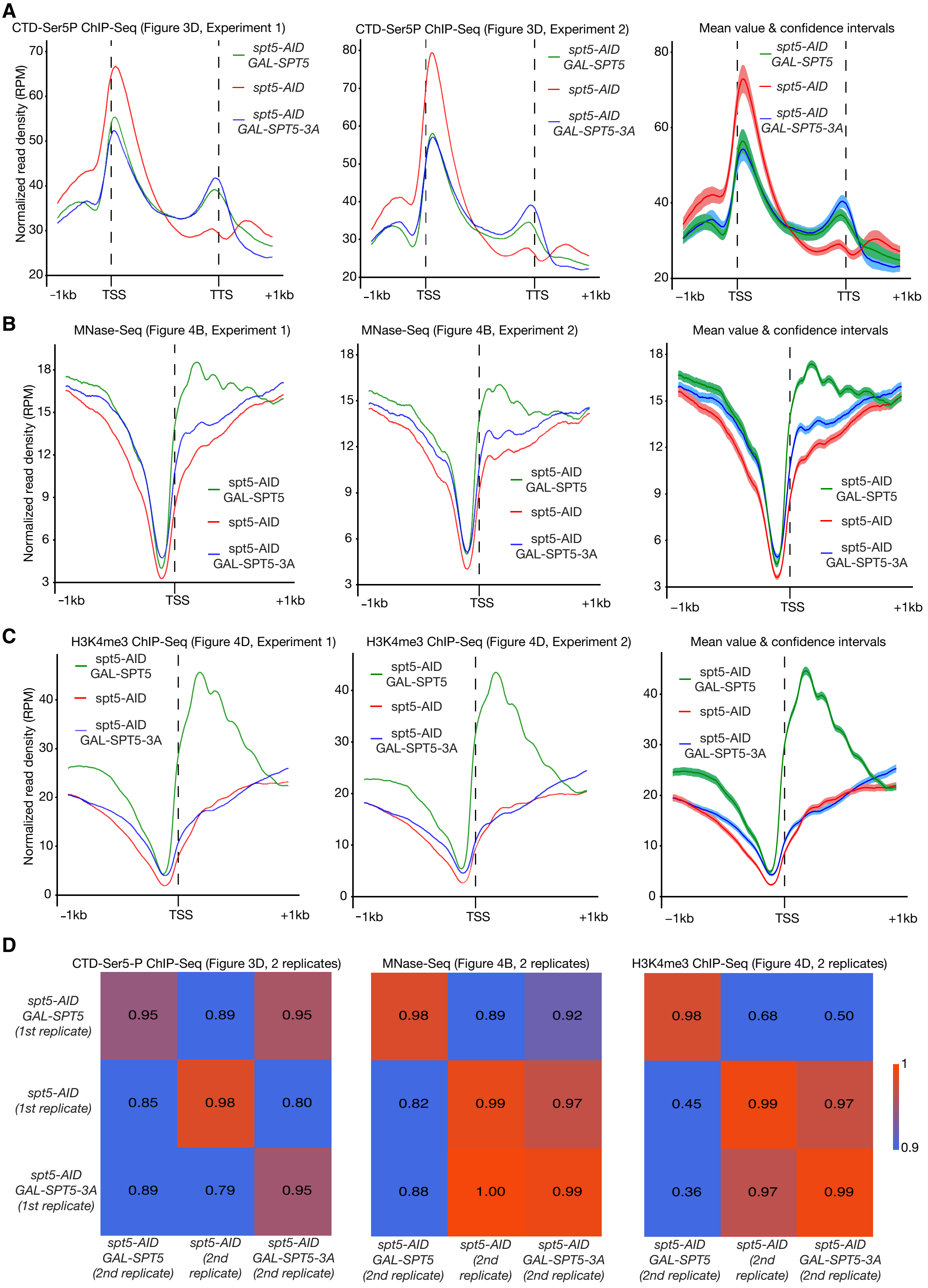

Figure EV3.

© 2022 The Authors

Figure EV4. Spt5N histone binding activity is most important at highly expressed genes.

A Further examples of MNase-Seq data for the experiment presented in Fig 4A and B. The data correspond to two highly expressed genes (*COR1* and *KAR2*), two low expressed genes (*PKC1* and *VPS72*) and two inactive genes (*PHO89* and *YOR389W*), based on the ChIP-Seq data for Rpb1 CTD-Ser5P in Fig 3.

B Analogous data for the same six genes, from the H3K4me3 ChIP-Seq experiment in Fig 4C and D.

© 2022 The Authors

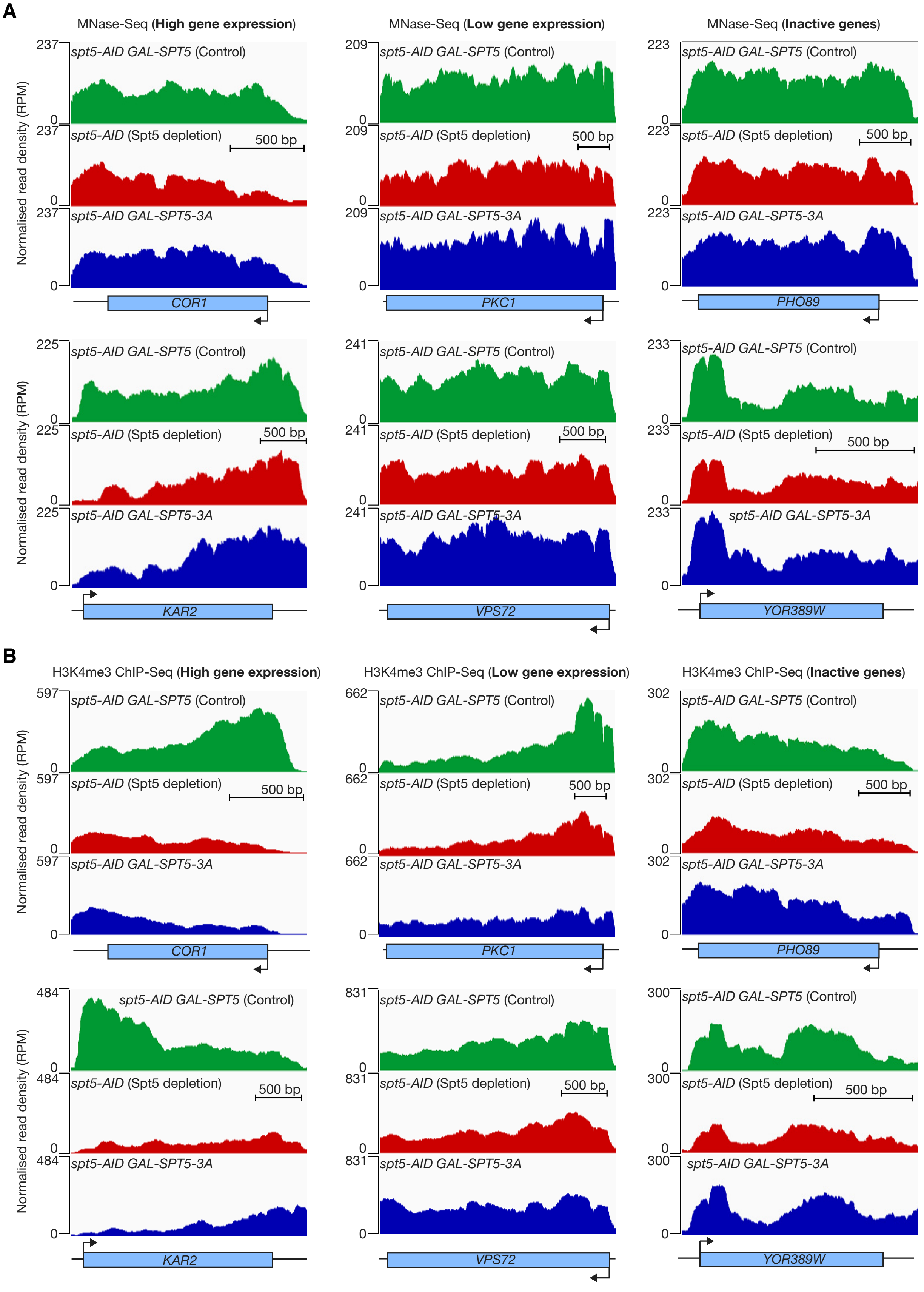

Figure EV4.

© 2022 The Authors
