# Supplementary material for: Spt5 histone binding activity preserves chromatin during transcription by RNA polymerase II
Source: EMBO J. 2022 Feb 1;41(5):e109783. doi: 10.15252/embj.2021109783 (PMC8886531; doi:10.15252/embj.2021109783)
Supplement: Supplementary file 2 — Table EV1 [file EMBJ-41-e109783-s004.docx]

**Table EV1:** reagents used in this study

| **REAGENT or RESOURCE** | **Source** | **Identifier** | **Additional information** |
| --- | --- | --- | --- |
| **Antibodies** | | |  |
| Anti-Sheep IgG  Coupled to Dynabeads M-270 Epoxy magnetic beads for immunoprecipitation experiments | Sigma-Aldrich | S1265 | Rabbit polyclonal antibody |
| Peroxidase Anti-Peroxidase soluble complex | Sigma-Aldrich | P1291 | Complex of peroxidase with anti-peroxidase antibodies produced in rabbit |
| Anti-Spt16  (antigen 729-950 *S. cerevisae*)  Dilution 1:1000 for immunoblotting | Foltman et al, 2013 | N/A | Sheep polyclonal antibody |
| Anti-Pob3  (antigen 176-397 *S. cerevisae*)  Dilution 1:1000 for immunoblotting | Foltman et al, 2013 | N/A | Sheep polyclonal antibody |
| Anti-histone H2A  Dilution 1:2000 for immunoblotting | Active Motif | 39235 | Rabbit polyclonal antibody |
| Anti-histone H2B  Dilution 1:1000 for immunoblotting | Active Motif | 39237 | Rabbit polyclonal antibody |
| Anti-histone H3  Dilution 1:500 for immunoblotting | This study | N/A | Sheep polyclonal antibody |
| Anti-histone H4  Dilution 1:1000 for immunoblotting | abcam | Ab10158 | Rabbit polyclonal antibody |
| Anti-Mcm2  (antigen 1-222 *S. cerevisae*)  Dilution 1:1000 for immunoblotting | Foltman et al, 2013 | N/A | Sheep polyclonal antibody |
| Anti-Spt5  (antigen 792-1013 *S. cerevisae*)  Dilution 1:1000 for immunoblotting | MRC PPU Reagents & Services | SA016 | Sheep polyclonal antibody |
| Anti-RNA Pol II CTD Ser5P  Dilution 1:4000 for immunoblotting | Active Motif | 61086 | Rat monoclonal antibody |
| Anti-RNA Pol II CTD Ser2P  Dilution 1:4000 for immunoblotting | Active Motif | 61084 | Rat monoclonal antibody |
| Anti-sheep IgG - Peroxidase  Dilution 1:10000 for immunoblotting | Sigma-Aldrich | A3415 | Donkey polyclonal secondary antibody |
| Anti-rabbit IgG - Peroxidase  Dilution 1:10000 for immunoblotting | Sigma-Aldrich | A6667 | Goat polyclonal secondary antibody |
| Anti-rat IgG - Peroxidase  Dilution 1:10000 for immunoblotting | Sigma-Aldrich | A9037 | Goat polyclonal secondary antibody |
| Anti-RNA Pol II CTD Ser5P | Millipore | 04-1572 | Rat monoclonal antibody |
| Anti-H3K4me3  Dilution 1:2000 for immunoblotting | Abcam | Ab8580 | Rabbit polyclonal antibody |
| Anti-AID  Dilution 1:1000 for immunoblotting | MRC PPU Reagents & Services | S880D | Sheep polyclonal antibody |
|  | | |  |
| **Chemicals, Peptides, and Recombinant Proteins** | | |  |
| Nocodazole | Sigma-Aldrich | M1404 | N/A |
| α-factor | EZBiolab | N/A | Custom synthesis |
| 3-indoleacetic acid (IAA) | Sigma Aldrich | I3750-5G-A | N/A |
| Roche Complete EDTA-free protease inhibitor cocktail | Roche | 11873580001 | N/A |
| Sigma protease inhibitor cocktail | Sigma-Aldrich | P8215 | N/A |
| SIGMAFAST protease inhibitor cocktail | Sigma-Aldrich | S8830 | N/A |
| Universal nuclease | ThermoFisher Scientific | 123991963 | N/A |
| MNase | Worthington Biochemical | 9013-53-0 | N/A |
| Dynabeads M-270 Epoxy | Life Technologies | 14302D | N/A |
| NuPage Novex 4-12% Bis-Tris gels | ThermoFisher Scientific | WG1402BOX | N/A |
| Simply Blue SafeStain | ThermoFisher Scientific | LC6060 | N/A |
| 1X Halt Protease Inhibitor cocktail | ThermoFisher Scientific | PN78439 | N/A |
| Glutathione Sepharose 4B | ThermoFisher Scientific | 17-0756-01 | N/A |
| Protein G Sepharose 4 Fast Flow | GE | 17-0618-02 | N/A |
| StrepTactin Superflow | IBA Lifesciences | 2-1206-025 | N/A |
| Ni-NTA agarose | Qiagen | 30210 | N/A |
| Chelex-100 | Biorad | 142-1253 | N/A |
|  | | | |
| ***E. coli* strain** | | | |
| Rosetta (DE3) pLysS | Novagen | 70956 | N/A |
|  | | |  |
| **Budding yeast strains** | | |  |
| *S. cerevisiae*: Strain W303-1  *MAT***a** *ade2-1 ura3-1 his3-11,15 trp1-1 leu2-3,112 can1-100 / MATα ade2-1 ura3-1 his3-11,15 trp1-1 leu2-3,112 can1-100* | N/A | N/A |  |
| *S. cerevisiae*: Strain W303-1a  *MAT***a** *ade2-1 ura3-1 his3-11,15 trp1-1 leu2-3,112 can1-100* | N/A | N/A | Figures 2C, 2D |
| *S. cerevisiae*: Strain YCE36  *MAT***a** *pep4Δ::ADE2 ura3-1::pRS306-GAL-ScSpt5 1-285-ProteinA-7His (URA3)* | This study | N/A | Figures 1F, 1G, 1H, 2B, 2D, S1A |
| *S. cerevisiae*: Strain YCE37  *MAT***a** *pep4Δ::ADE2 ura3-1::pRS306-GAL-ScSpt5 1-1063-ProteinA-7His (URA3)* | This study | N/A | Figures 2B, 2D |
| *S. cerevisiae*: Strain YCE47  *MAT***a** *pep4Δ::ADE2 ura3-1::pRS306-GAL-ScSpt5 267-1063-ProteinA-7His (URA3)* | This study | N/A | Figures 2B, 2D |
| *S. cerevisiae*: Strain YCE121  *MAT***a** *pep4Δ::ADE2 ura3-1::pRS306-GAL-ScSpt5 1-223-ProteinA-7His (URA3)* | This study | N/A | Figure S1A |
| *S. cerevisiae*: Strain YCE122  *MAT***a** *pep4Δ::ADE2 ura3-1::pRS306-GAL-ScSpt5 1-277-ProteinA-7His (URA3)* | This study | N/A | Figure S1A |
| *S. cerevisiae*: Strain YCE123  *MAT***a** *pep4Δ::ADE2 ura3-1::pRS306-GAL-ScSpt5 46-285-ProteinA-7His (URA3)* | This study | N/A | Figure S1A |
| *S. cerevisiae*: Strain YCE124  *MAT***a** *pep4Δ::ADE2 ura3-1::pRS306-GAL-ScSpt5 97-285-ProteinA-7His (URA3)* | This study | N/A | Figure S1A |
| *S. cerevisiae*: Strain YCE130  *MAT***a** *pep4Δ::ADE2 ura3-1::pRS306-GAL-ScSpt5 97-223-ProteinA-7His (URA3)* | This study | N/A | Figure S1A |
| *S. cerevisiae*: Strain YCE132  *MAT***a** *pep4Δ::ADE2 ura3-1::pRS306-GAL-ScSpt5 1-285-3A-ProteinA-7His (URA3) [3A=F180A, E184A, V187A]* | This study | N/A | Figures 1F, 1G, 1H, 2D, S1A |
| *S. cerevisiae*: Strain YCE141  *MAT***a** *pep4Δ::ADE2 ura3-1::pRS306-GAL-ScSpt5 1-1063-3A-ProteinA-7His (URA3) [3A=F180A, E184A, V187A]* | This study | N/A | Figures 2B, 2D |
|  |  |  |  |
| *S. cerevisiae*: Strain YCE281  *MAT***a** *spt5-aid ura3-1:: ADH1-OsTIR1-9MYC (URA3 and K.l.TRP1)* | This study | N/A | Figures 2A, 3B, 3C, 3D, 3F, 3G, 4A, 4B, 4C, 4D, 4E |
| *S. cerevisiae*: Strain YCE350  *MAT***a** *spt5-aid ura3-1:: ADH1-OsTIR1-9MYC (URA3 and K.l.TRP1) leu2-3::pRS305-GAL-ScSpt5 1-1063-ProteinA-7His (LEU2)* | This study | N/A | Figure 2C |
| *S. cerevisiae*: Strain YCE356  *MAT***a** *spt5-aid ura3-1:: ADH1-OsTIR1-9MYC (URA3 and K.l.TRP1) leu2-3::pRS305-GAL-ScSpt5 1-1063-3A-ProteinA-7His (LEU2) 3A=F180A, E184A, V187A* | This study | N/A | Figure 2C |
| *S. cerevisiae*: Strain YCE421  *MAT***a** *spt5-aid ura3-1:: ADH1-OsTIR1-9MYC (URA3 and K.l.TRP1) leu2-3::pRS305-GAL-ScSpt5 1-285-ProteinA-7His (LEU2)* | This study | N/A | Figure 2C |
| *S. cerevisiae*: Strain YCE423  *MAT***a** *spt5-aid ura3-1:: ADH1-OsTIR1-9MYC (URA3 and K.l.TRP1) leu2-3::pRS305-GAL-ScSpt5 267-1063-ProteinA-7His (LEU2)* | This study | N/A | Figure 2C |
| *S. cerevisiae*: Strain YCE761  *MAT***a** *pep4Δ::ADE2 ura3-1::pRS306-GAL-ScSpt5 97-202-ProteinA-7His (URA3)* | This study | N/A | Figure S1A |
| *S. cerevisiae*: Strain YCE763  *MAT***a** *pep4Δ::ADE2 ura3-1::pRS306-GAL-ScSpt5 112-223-ProteinA-7His (URA3)* | This study | N/A | Figure S1A |
| *S. cerevisiae*: Strain YCE765  *MAT***a** *pep4Δ::ADE2 ura3-1::pRS306-GAL-ScSpt5 144-223-ProteinA-7His (URA3)* | This study | N/A | Figure S1A |
| *S. cerevisiae*: Strain YCE1063  *MAT***a** *pep4Δ::ADE2 ura3-1::pRS306-GAL-SpSpt5 1-240-ProteinA-7His (URA3)* | This study | N/A | Figures 1H, S1B |
| *S. cerevisiae*: Strain YCE1065  *MAT***a** *pep4Δ::ADE2 ura3-1::pRS306-GAL-SpSpt5 1-240-3A-ProteinA-7His (URA3) [3A=F146A E150A V153A]* | This study | N/A | Figures 1H, S1B |
| *S. cerevisiae*: Strain YCE1101  *MAT***a** *pep4Δ::ADE2 ura3-1::pRs306-GAL-SpSpt5 80-180-ProteinA-7His (URA3)* | This study | N/A | Figure S1B |
| *S. cerevisiae*: Strain YCE1111  *MAT***a** *ura3-1:: ADH1-OsTIR1-9MYC (URA3 and klTRP1)* | This study | N/A | Figures 2A, 2C |
| *S. cerevisiae*: Strain YCE1140  *MAT***a** *ura3-1:: ADH1-OsTIR1-9MYC (URA3 and klTRP1) leu2-3::pRS305-GAL-ScSpt5 1-1063 (LEU2)* | This study | N/A | Figures 2C, 3B, 3C, 3D, 3F, 3G, 4A, 4B, 4C, 4D, 4E, 4F |
| *S. cerevisiae*: Strain YCE1141  *MAT***a** *ura3-1:: ADH1-OsTIR1-9MYC (URA3 and klTRP1) leu2-3::pRS305-GAL-ScSpt5 1-1063-3A (LEU2)*  *[3A=F180A, E184A, V187A]* | This study | N/A | Figures 2C, 3B, 3C, 3D, 3F, 3G, 4A, 4B, 4C, 4D, 4E, 4F |
| *S. cerevisiae*: Strain YMP598-4  *MAT***a** *pep4Δ::ADE2 SPT5-9MYC (HIS3MX)* |  | N/A | Figure 2E |
| *S. cerevisiae*: Strain YSS3  *MAT***a** *pep4Δ::ADE2* |  | N/A | Figure S1B |
|  | | | |
| **Plasmid DNA** | | | |
| pCDFduet.H2A-H2B | Kingston et al 2011 | Martin Singleton, Francis Crick Institute (London) | Expression in bacteria |
| pETduet.H3-H4 | Kingston et al 2011 | Martin Singleton, Francis Crick Institute (London) | Expression in bacteria |
| pET413c (GST-6HIS) | This study | DU70779 | Expression in bacteria |
| pCE161 (pET41c-GST-HsMcm2 43-160-StreptagIII) | This study | DU70707 | Expression in bacteria |
| pCE223 (pET41c-GST-HsMcm2 43-160-2A-StreptagIII)  [2A = Y274A Y283A] | This study | DU70711 | Expression in bacteria |
| pCE122 (pET41c- GST-ScSpt5 97-223-8HIS) | This study | DU70712 | Expression in bacteria |
| pCE131 (pET41c-GST-ScSpt5 97-223-3A-8HIS)  [3A = F180A, E184A, V187A] | This study | DU70710 | Expression in bacteria |
| pCE167 (pET41c-GST-SpSpt5 80-180-StreptagIII) | This study | DU70708 | Expression in bacteria |
| pCE1 (pRS306-GAL-ScSpt5 1-1063-ProteinA-7HIS) | This study | DU70703 | Integration at *ura3* locus for expression in yeast |
| pCE2 (pRS306-GAL-ScSpt5 1-285-ProteinA-7HIS) | This study | DU70686 | Integration at *ura3* locus for expression in yeast |
| pCE3 (pRS306-GAL-ScSpt5 267-1063-ProteinA-7HIS) | This study | DU70687 | Integration at *ura3* locus for expression in yeast |
| pCE15 (pRS306-GAL-ScSpt5 1-223-ProteinA-7HIS) | This study | DU70688 | Integration at *ura3* locus for expression in yeast |
| pCE16 (pRS306-GAL-ScSpt5 1-277-ProteinA-7HIS) | This study | DU70689 | Integration at *ura3* locus for expression in yeast |
| pCE17 (pRS306-GAL-ScSpt5 46-285-ProteinA-7HIS) | This study | DU70690 | Integration at *ura3* locus for expression in yeast |
| pCE18 (pRS306-GAL-ScSpt5 97-285-ProteinA-7HIS) | This study | DU70702 | Integration at *ura3* locus for expression in yeast |
| pCE24 (pRS306-GAL-ScSpt5 97-223-ProteinA-7HIS) | This study | DU70701 | Integration at *ura3* locus for expression in yeast |
| pCE25 (pRS306-GAL-ScSpt5 1-285-3A-ProteinA-7HIS)  [3A = F180A, E184A, V187A] | This study | DU70691 | Integration at *ura3* locus for expression in yeast |
| pCE35 (pRS306-GAL-ScSpt5-3A-ProteinA-7HIS)  [3A = F180A, E184A, V187A] | This study | DU70692 | Integration at *ura3* locus for expression in yeast |
|  |  |  |  |
| pCE73 (pRS306-GAL-SpSpt5 1-240-ProteinA-7HIS) | This study | DU70715 | Integration at *ura3* locus for expression in yeast |
| pCE74 (pRS306-GAL-SpSpt5 1-240-3A-ProteinA-7HIS)  [3A = F146A, E150A, V153A] | This study | DU70714 | For expression in yeast |
| pCE77 (pRS306-own promoter-ScSpt5 1-1063) | This study | DU70700 | Integration at *ura3* locus for expression in yeast |
| pCE108 (pRS306-own promoter-ScSpt5 1-1063-3A)  [3A = F180A, E184A, V187A] | This study | DU70704 | Integration at *ura3* locus for expression in yeast |
| pCE79 (pRS305-GAL-ScSpt5-ProteinA-7HIS) | This study | DU70693 | Integration at *leu2* locus for expression in yeast |
| pCE80 (pRS305-GAL-ScSpt5-3A-ProteinA-7HIS)  [3A = F180A, E184A, V187A] | This study | DU70694 | Integration at *leu2* locus for expression in yeast |
| pCE106 (pRS305-GAL-ScSpt5 1-285-ProteinA-7HIS) | This study | DU70695 | Integration at *leu2* locus for expression in yeast |
| pCE107 (pRS305-GAL-ScSpt5 267-1063-ProteinA-7HIS) | This study | DU70696 | Integration at *leu2* locus for expression in yeast |
| pCE123 (pRS306-GAL-ScSpt5 97-202-ProteinA-7HIS) | This study | DU70697 | Integration at *ura3* locus for expression in yeast |
| pCE124 (pRS306-GAL-ScSpt5 112-223-ProteinA-7HIS) | This study | DU70698 | Integration at *ura3* locus for expression in yeast |
| pCE126 (pRS306-GAL-ScSpt5 144-223-ProteinA-7HIS) | This study | DU70699 | Integration at *ura3* locus for expression in yeast |
| pCE159 (pRS306-GAL-SpSpt5 80-180-ProteinA-7HIS) | This study | DU70713 | Integration at *ura3* locus for expression in yeast |
| pCE169 (pRS305-GAL-ScSpt5 1-1063) | This study | DU70706 | Integration at *leu2* locus for expression in yeast |
| pCE170 (pRS305-GAL-ScSpt5 1-1063-3A)  [3A = F180A, E184A, V187A] | This study | DU70705 | Integration at *leu2* locus for expression in yeast |
|  | | | |
| **DNA oligonucleotides** | | | |
| 5016  TACTGAgctagcATGAGTGACAACTCGGACACAAACGTGAG | This study | N/A | Forward primer for construction of pCE1, pCE2, pCE25, pCE169, pCE170 |
| 5017  tgatcaGGATCCgtcgacctgcagcgtacgATGACCTCCCCATGTACTGTTACCACCATAG | This study | N/A | Reverse primer for construction of pCE1, pCE3 |
| 5015  gagctaGGATCCgtcgacctgcagcgtacgAGCTGTATCAACACTTGGTAGGAGAAACCTC | This study | N/A | Reverse primer for construction of pCE2, pCE17, pCE18, pCE25 |
| 5018  tgactagctagcATGGCTGCTCAAGATGGTTACGTGCCCCAGAG | This study | N/A | Forward primer for construction of pCE3 |
| 5331  TACTGAtctagaATGAGTGACAACTCGGACACAAACGTGAG | This study | N/A | Forward primer for construction of pCE15, pCE16 |
| 5299  tgatcaGGATCCgtcgacctgcagcgtacgTGCGCCTGGAGCACTTGCTTCGTCATC | This study | N/A | Reverse primer for construction of pCE15, pCE24, pCE124, pCE126 |
| 5300  tgatcaGGATCCgtcgacctgcagcgtacgAAACCTCTGGGGCACGTAACCATCTTG | This study | N/A | Reverse primer for construction of pCE16 |
| 5301  TACTGAtctagaATGGTGACCACAACGGAAAGTACAGAAC | This study | N/A | Forward primer for construction of pCE17 |
| 5302  TACTGAtctagaATGGCTACAGATGATGCCCAAGCAACTTTG | This study | N/A | Forward primer for construction of pCE18, pCE24, pCE123 |
| 5542  CAAagcTCTGTTCCTCTCCTGACGACG | This study | N/A | Reverse primer to introduce 3A mutations in pCE25 |
| 5543  CGTCGTCAGGAGAGGAACAGAgctTTGGATATTgcaGCTGAGgctAGTGATGATGAAGATGAAGATGAAG | This study | N/A | Forward primer to introduce 3A mutations in pCE25 |
| 6049  tactgaGCGGCCGCtgaaaaaaaactcggaaggtatttaggtgaccttgcggcttcttaatgctg | This study | N/A | Forward primer for construction of pCE77, pCE108 |
| 6050  gagctaGGATCCgcatacatacatacatacgtatatgtaaaagttagaatcaaggaaaactc | This study | N/A | Reverse primer for construction of pCE77, pCE108 |
| 6709  tgatcaGGATCCgtcgacctgcagcgtacgCAACTCTGAATCCTCTTCATCTTCATC | This study | N/A | Reverse primer for construction of pCE123 |
| 6707  TACTGAtctagaATGGCAAATGAAATTGTCAAGAAGGAAGAG | This study | N/A | Forward primer for construction of pCE124 |
| 6708  TACTGAtctagaATGGGCGATAACAAAGATGAAGACGATGATG | This study | N/A | Forward primer for construction of pCE126 |
|  |  |  |  |
|  |  |  |  |
|  |  |  |  |
| 7419  TACTGAactagtATGGGGAATGAAAACGAAGTCGATAATG | This study | N/A | Forward primer for construction of pCE159 |
| 7420  gagctaGGATCCgtcgacctgcagcgtacgCGCTCCAACTTCTTCTTCAATGAAACCATC | This study | N/A | Reverse primer for construction of pCE159 |
| 8401  tgatcaGGATCCttaATGACCTCCCCATGTACTGTTACCACCATAG | This study | N/A | Reverse primer for construction of pCE169, pCE170 |
| 6705  tcatgaACTAGTgaaaacctgtattttcagggcggatcaGCTACAGATGATGCCCAAGCAACTTTG | This study | N/A | Forward primer for construction of pCE122, pCE131 |
| 6706  TAGTACctcgagTGCGCCTGGAGCACTTGCTTCGTCATC | This study | N/A | Reverse primer for construction of pCE122, pCE131 |
|  |  |  |  |
|  |  |  |  |
| 7568  TACTGAgctagcGGGAATGAAAACGAAGTCGATAATG | This study | N/A | Forward primer for construction of pCE167 |
| 7569  GAGCTAggatccCGCTCCAACTTCTTCTTCAATGAAACCATC | This study | N/A | Reverse primer for construction of pCE167 |
| 7546  TACTGAgctagcGGTAGAGATTTGCCACCTTTCG | This study | N/A | Forward primer for construction of pCE161, pCE223 |
| 7547  GAGCTAggatccATCTTCAGTAGCTCTCTCAAC | This study | N/A | Reverse primer for construction of pCE161, pCE223 |
