# Supplementary material for: Spt5 histone binding activity preserves chromatin during transcription by RNA polymerase II
Source: EMBO J. 2022 Feb 1;41(5):e109783. doi: 10.15252/embj.2021109783 (PMC8886531; doi:10.15252/embj.2021109783)
Supplement: Supplementary file 4 [file EMBJ-41-e109783-s002.pdf]

THE EMBO JOURNAL

Spt5 histone binding activity preserves chromatin during transcription by RNA polymerase II

Cecile Evrin, Albert Serra-Cardona, Shoufu Duan, Progya Mukherjee, Zhiguo Zhang, and Karim Labib
DOI: 10.15252/embj.2021109783

Corresponding author(s): Karim Labib (kpmlabib@dundee.ac.uk) , Zhiguo Zhang (zz2401@cumc.columbia.edu)

Review Timeline:

Submission Date:	22nd Sep 21
Editorial Decision:	20th Oct 21
Revision Received:	12th Dec 21
Editorial Decision:	21st Dec 21
Revision Received:	22nd Dec 21
Accepted:	6th Jan 22

Editor: Hartmut Vodermaier

Transaction Report:

(Note: With the exception of the correction of typographical or spelling errors that could be a source of ambiguity, letters and reports are not edited. Depending on transfer agreements, referee reports obtained elsewhere may or may not be included in this compilation. Referee reports are anonymous unless the Referee chooses to sign their reports.)

1st Editorial Decision 20th Oct 2021

Thank you again for submitting your study on Spt5 histone binding during transcription for our consideration. It has now been assessed by four expert referees, whose reports are copied below. All reviewers appreciate the potential interest of your functional/in vivo results, and their timeliness in light of recent structural work. We shall therefore be happy to consider this study further for publication in The EMBO Journal, pending satisfactory revision along the lines of the enclosed comments. As you will see, the issues and suggestions brought forward by the referees are mostly aimed at strengthening the experimental data, improving their presentation, and deepening the understanding of a few particular aspects.

Detailed information on preparing, formatting and uploading a revised manuscript can be found below and in our Guide to Authors. Please be reminded that it is our policy to allow only a single round of (major) revision, making it important to carefully respond to all points raised at this point. Should this require more time than our default three-months revision period, we would be open to offering an extension, during which our 'scooping protection' would of course remain valid.

REFEREE REPORTS

Referee #1:

In this interesting paper, the authors present experiments that show that the Spt5 N-terminal end can bind histones. Furthermore a mutation that alters 3 amino acids (Spt5-3A) abolishes this binding and causes a dominant-negative phenotype in vivo, suggesting that this aspect of Spt5 function is critical. Using a conditional depletion/expression system, the authors then go on to provide evidence that the Spt5-3A mutant does not impair transcription elongation, but that it does disrupt chromatin structure. While there have been some other recent studies that have implicated Spt5 with chromatin structure, this work provides the first in vivo evidence, thus making a nice contribution to the field that will be of general interest. Below are a number of comments, about controls that should be added and more information that should be provided about the experiments.

1. page 8, lines 168-172 - It might also be appropriate to cite Ehara et al. 2019, as they proposed that the Spt5 N-terminal end might interact with nucleosomes.

2. Intro - The authors have written a very nice review in the Intro, with excellent citation of the relevant literature. However, they have overlooked one contribution that addressed the role of Spt4/5 in overcoming nucleosomal barriers - Crickard et al. (2017) NAR 45, 6362. Citation of this work, where relevant, should be added.

3. Figure 1 - What is known about the truncations of GST-Spt5? Do they still have the GST tag? Is there any knowledge of whether they bind to histones?

4. Figure 3 and accompanying text on pages 12-13 - The results for ChIP-seq of Ser5P are interesting. However, I'm wondering about the choice of looking at only Ser5P, as that looks only at a subpopulation of all of the elongating RNAPII. Wouldn't it be better to look at total RNAPII or to include Ser2P? Most studies that have looked at Spt5 transcription defects see more pronounced effects further downstream.

5. Figure 3B - From the Western analysis, it appears that the GAL-SPT5 construct overexpresses SPT5. Previous work has shown that overexpression of SPT5 causes a mutant phenotype (Swanson et al., 1991, MCB 11, 3009). Therefore, overexpression might affect the results that are obtained with this experimental protocol. A good control would be to test a wild-type strain and compare it to the SPT5-AID/GAL-SPT5 strain.

6. Related to their system for analysis of Spt5, while the Western analysis shows strong depletion, it would be strengthen the

analysis to show that the Spt5 ChIP signal is also depleted via ChIP-seq. And it would also be very relevant to analyze the Spt5-ChIP seq signal after GAL induction for both wt and the 3A mutant. This could be done using different epitope tags on the genomic and GAL-induced copies of Spt5.

7. Also for Figure 3 and Figure 4 - what was the cell viability after cell cycle arrest, IAA addition, and expression of the Spt5 mutant? It is quite important to know that these steps are not causing inviability, particularly since there is depletion of an essential protein and expression of a dominant-negative protein.

8. For all ChIP-seq and MNase-seq experiments - please state in methods how many biological replicates were done (it sounds like it was two), as well as the reproducibility between the replicates. Then, for the metagene plots (Figures 3D, 4B, 4D) please state explicitly if we are looking at the average of the two independent experiments (as it does for 4D). Also, please plot these values showing the confidence intervals.

9. Figure 3D - The level of Ser5P appears to be lower in the SPT5-3A strain. Can the authors comment on this? It's possible that no strong conclusion can be made due to the absence of spike-in normalization; however, given that there are effects seen later in the MNase-seq and H3K4me3 ChIP-seq experiments, it is of great interest to know if there is any correlation of effects on transcription with these later results. For example, since the H3K4me3 effects were greatest for the most highly transcribed quartile, what happens with the Ser5P profile if it is divided into quartiles? Given that the Spt5N-3A mutant fails to bind histones, it is reasonable to assume that its primary defect in vivo is on chromatin structure, but it cannot be ruled out that its primary effect is on transcription, with changes in chromatin structure as a consequence.

10. In Methods, the authors explain that their data analysis is performed on 821 transcriptionally active genes. This aspect of the analysis should be presented in results, as most omics analyses would include a larger number of genes. They should also make this clear in the figure legends.

11. For all ChIP-seq and MNase-seq experiments, Western analysis should be presented to test whether the proteins being assayed have normal levels. This is clearly relevant for the Ser5P signal after Spt5 depletion, given the two recent studies that showed that Spt5 depletion causes depletion of RNAPII (Aoi et al. and Hu et al., Mol. Cell). Also, for the MNase-seq experiments, it's conceivable that there is a reduced level of histones in the spt5-AID samples where the signal is reduced. The Western in 3B suggests that there is a normal level of H3K4me3, but that Western did not include total levels of histone H3, nor did it include either a good loading control or statistical analysis.

Referee #2:

In this manuscript, Ervin, Serra-Caradona et al. show that the highly conserved and essential Spt5 transcription elongation factor contains a histone-binding motif that is required for cell viability. The authors further show that this domain is required to prevent the loss of nucleosomal histones and maintenance of histone H3K4me3 in actively transcribed genes. The findings establish a role for Spt5 in histone transfer in addition to its previously described role in processive transcription. This is an excellent paper that provides new insight into the biochemical functions of a highly conserved transcription-associated protein. The new insights about potential parallels in mechanisms of histone capture and transfer during transcription involving similar roles for Spt5 and Mcm2 during transcription and replication, respectively, are very interesting. The results are mostly straightforward and support the key conclusions of the paper.

Specific comments

1. The authors conclude based on MNase-seq results that Spt5 is required for maintenance of nucleosomal histones in transcribed regions. The analysis of the MNase-seq results seems incomplete. In Figure 4A, they show an example of nucleosome loss at the YFL026W genes after Spt5-AID depletion. They show provide examples of the effect of Spt5 depletion at a transcriptionally active versus lowly transcribed or transcriptionally silent (such as the silent mating type loci) genes to test their hypothesis that Spt5 is required to maintain nucleosomes during transcription. The model is of course very reasonable, but as the data are presented, direct support for transcription coupled loss of nucleosomes is not included.

2. In Figure 3A-D, the authors present data on the effect of Spt5 depletion and the inability of GAL-SPT5-3A histone binding mutant to rescue nucleosome loss observed after Spt5 depletion. They should also include data for the expected rescue of Spt5 depletion by wild-type GAL-SPT5 (as in Figure 2E). Otherwise, it's difficult to conclude that the observed loss of nucleosomes and H3K4me3 are caused by SPT5-3A loss of histone binding (as opposed to GAL overexpression).

Minor
1. It would be preferable to track histone loss using a tag exchange strategy, but I am persuaded with the authors use of H3K4me3 as a proxy for old histones.

2. Have the authors tested whether the non-essential SPT4 affects nucleosome maintenance. Seems unlikely in light of the essential function of the histone binding of Spt5.

Referee #3:

Evrin et al. provide extensive evidence indicating that Spt5 has a histone-binding domain that is selective for H3-H4, that this domain is essential for viability in yeast, and that it is also required to maintain normal nucleosome occupancy and modification states downstream of transcription start sites. They show that histone binding is not an essential part of the elongation function of Spt5, as active Pol II occupancy within transcription units remained relatively normal after cells were forced to rely on the mutant form lacking the binding domain. This results in a very interesting suggestion that Spt5 is a key part of the histone chaperoning chain that also involves Spt6 and FACT during transcription, which is in line with the recent structures from the Cramer lab. The data are of high quality and answer some long-standing questions while also adding new pieces to the puzzle regarding how polymerases manage to progress through chromatin and then restore its properties. This story will be of broad interest to a wide audience. Some additional analysis and discussion could enhance the manuscript, as described below.

1) The correlation between the effects of the spt5N-3A mutation and transcription frequency on H3K4me3 depopulation in Fig 4F was surprisingly small, but this may have to do with the way the data are presented. Instead of plotting occupancy in the mutant against the WT, perhaps the fold change in mutant/WT plotted against transcription would be more striking?

2) I was unable to find any discussion regarding how the residues were chosen for mutation in the spt5N-3A mutation. The acidic region with embedded hydrophobic residues is reminiscent of the interaction of FACT with H2A-H2B and the residues mutated look like they might be based on the same principles. However, interactions with H3-H4 are expected to be distinct as H3-H4 makes more extensive and stronger contact with DNA than H2A-H2B does. It would therefore be interesting to others in the field to know what else the authors tried or how they settled on the residues chosen to see if general principles can be extracted.

3) In line 143, the Endnote citation was not converted to a reference as intended.

Referee #4:

This study identified a histone binding motif at the Spt5 proteins and found that this histone binding ability contributes to chromatin integrity during gene transcription. They found that Spt5-N terminus can bind H3-H4 in vitro and this motif is required for cell's viability. They also found that mutations at this binding site led to a nucleosomal histone loss during gene transcription. Therefore, they proposed a new role of Spt5 in capture and re-deposition histones during gene transcription. In general, this study is well controlled and provides novel information regarding molecular mechanisms of preserving chromatin during gene transcription. To further clarify this mechanism, it would be better to perform the following tests:

1, As the authors mentioned, Spt5N and FACT can bind simultaneously to histones released from chromatin. IP results also showed that Spt5N-3A mutant proteins lost the interactions with histones, as well as with FACT proteins. Is this suggested that the Spt5-FACT interaction is bridged by H3-H4? Whether the FACT bound histones is reduced in spt5-3A mutant cells?

2. Since FACT is also involved in preventing nucleosomal histone H3-H4 loss during gene transcription, it would be better to check the status of FACT association at gene body regions.

We thank all three referees for their helpful comments and for their appreciation of our work. In response to the points raised by the referees, we now include additional & revised data in Figure 3D, Figure 4F, Figure EV2, Figure EV3, Appendix Figure S1 and a 'Figure for referees only'. The changes that we have made are described below and are also highlighted in the revised manuscript in red text.

Please note: in order to access the raw and analyzed sequencing data (deposited in the NCBI under the GEO accession number), please go to: https://www.ncbi.nlm.nih.gov/geo/query/acc.cgi?acc=GSE181901 and use the token ebgjeowcvnqrlcp.

Referee #1:

In this interesting paper, the authors present experiments that show that the Spt5 N-terminal end can bind histones. Furthermore, a mutation that alters 3 amino acids (Spt5-3A) abolishes this binding and causes a dominant-negative phenotype in vivo, suggesting that this aspect of Spt5 function is critical. Using a conditional depletion/expression system, the authors then go on to provide evidence that the Spt5-3A mutant does not impair transcription elongation, but that it does disrupt chromatin structure. While there have been some other recent studies that have implicated Spt5 with chromatin structure, this work provides the first in vivo evidence, thus making a nice contribution to the field that will be of general interest. Below are a number of comments, about controls that should be added and more information that should be provided about the experiments.

1. page 8, lines 168-172 - It might also be appropriate to cite Ehara et al. 2019, as they proposed that the Spt5 N-terminal end might interact with nucleosomes.

We now cite this paper on page 5, line 93.

2. Intro - The authors have written a very nice review in the Intro, with excellent citation of the relevant literature. However, they have overlooked one contribution that addressed the role of Spt4/5 in overcoming nucleosomal barriers - Crickard et al. (2017) NAR 45, 6362. Citation of this work, where relevant, should be added.

We now cite this paper on page 8, line 168.

3. Figure 1 - What is known about the truncations of GST-Spt5? Do they still have the GST tag? Is there any knowledge of whether they bind to histones?

The GST-Spt5 truncations in Figure 1 do indeed still have the GST tag (we know this from immunoblotting). We haven't studied these truncations in further detail, but the data summarised in Figure EV1A show which truncations of Spt5N are compatible with histone binding.

4. Figure 3 and accompanying text on pages 12-13 - The results for ChIP-seq of Ser5P are interesting. However, I'm wondering about the choice of looking at only Ser5P, as that looks only at a subpopulation of all of the elongating RNAPII. Wouldn't it be better to look at total RNAPII or to include Ser2P? Most studies that have looked at Spt5 transcription defects see more pronounced effects further downstream.

The approach suggested by the referee would indeed have been very comprehensive, but would not have changed the major conclusions of the manuscript. In choosing to do ChIP-Seq of Rpb1 CTD-Ser5P, we were guided in part by a previous study of Spt5 depletion in fission yeast (Shetty et al, 2017, Mol. Cell, 66, 77-88). This showed that the Rpb1 elongation defect upon Spt5 depletion

was equally apparent using ChIP-Seq of Rpb1 CTD-Ser5P, Rpb1 CTD-Ser2 or total Rpb1 (Figure S3B of Shetty et al, 2017). In our manuscript, we chose to use Rpb1 CTD-Ser5P as a representative mark of elongating Rpb1, and the defect seen upon depletion of Spt5-AID is quite comparable to that seen in fission yeast cells by Shetty et al (2017).

5. Figure 3B - From the Western analysis, it appears that the GAL-SPT5 construct overexpresses SPT5. Previous work has shown that overexpression of SPT5 causes a mutant phenotype (Swanson et al., 1991, MCB 11, 3009). Therefore, overexpression might affect the results that are obtained with this experimental protocol. A good control would be to test a wild-type strain and compare it to the SPT5-AID/GAL-SPT5 strain.

We appreciate the referee's point regarding previous data by Swanson & Winston (1991), which showed a weak 'spt phenotype' upon over-expression of SPT5.

Nevertheless, the data in our manuscript show that expression of GAL-SPT5 does not block cell growth and proliferation (Figure 2B, GAL-SPT5, 1-1063). Moreover, the experiments in Figures 3-4 directly compare the ability of GAL-SPT5 and GAL-SPT5-3A to rescue depletion of Spt5-AID. Therefore, the presented experiments already show the specific effect of the Spt5-3A mutations, independently of any effect of the altered level of Spt5 expression.

6. Related to their system for analysis of Spt5, while the Western analysis shows strong depletion, it would be strengthen the analysis to show that the Spt5 ChIP signal is also depleted via ChIP-seq. And it would also be very relevant to analyze the Spt5-ChIP seq signal after GAL induction for both wt and the 3A mutant. This could be done using different epitope tags on the genomic and GAL-induced copies of Spt5.

We take the referee's point, but unfortunately we did not have time for additional ChIP-Seq experiments within the timeframe for preparing the revised manuscript. We note that the data in Figure 2 show clearly that depletion of Spt5-AID is lethal and can be rescued by GAL-SPT5 but not by SPT5-3A. Moreover, Figures 3-4 show that depletion of Spt5-AID impairs RNAPII elongation – this elongation defect is complemented by expression of either GAL-SPT5 or GAL-SPT5-3A. In addition, parental chromatin is lost when GAL-SPT5-3A complements Spt5-AID depletion but not when Spt5-AID depletion is rescued by wild type GAL-SPT5. We hope the referee will agree that our data are already sufficiently robust.

7. Also for Figure 3 and Figure 4 - what was the cell viability after cell cycle arrest, IAA addition, and expression of the Spt5 mutant? It is quite important to know that these steps are not causing inviability, particularly since there is depletion of an essential protein and expression of a dominant-negative protein.

We now present new data in Appendix Figure S1B to address this issue. Although permanent depletion of Spt5-AID blocks cell growth (Figure 2E), the new data in Appendix Figure S1B show that the effects of transiently depleting Spt5-AID are reversible, when cells are placed on medium lacking auxin and the Spt5-AID protein is re-expressed.

It is important to note, however, that we already showed that the expression of Spt5-3A is dominant lethal, even when expressed from one copy of the endogenous SPT5 locus in a heterozygous SPT5 / SPT5-3A diploid cell (Figure 2A). Therefore, the data in our original manuscript clearly predicted that transient expression of GAL-SPT5-3A in spt5-AID cells should cause inviability, since the Spt5-3A protein would still be present when cells are transferred to medium lacking galactose and auxin. The new data in Appendix Figure S1B confirm this

prediction. These findings are discussed on lines 279-283 of the revised manuscript.

8. For all ChIP-seq and MNase-seq experiments - please state in methods how many biological replicates were done (it sounds like it was two), as well as the reproducibility between the replicates. Then, for the metagene plots (Figures 3D, 4B, 4D) please state explicitly if we are looking at the average of the two independent experiments (as it does for 4D). Also, please plot these values showing the confidence intervals.

In addition to the legends to Figures 3-4 that already stated that the ChIP-Seq and MNase-Seq data were from 'two independent experiments', we now specify in Materials and Methods that we did two biological replicates (pages 27-28, lines 645 and 670).

Regarding reproducibility between replicates, lines 709-713 ('Data Analysis of ChIP-Seq and MNase-Seq datasets' section of Materials and Methods) now states that "For the MNase-Seq, Rpb1 CTD-Ser5P ChIP and H3K4me3 ChIP datasets, the enrichment around the TSS of actively transcribed genes was calculated using computeMatrix from version 3.2.1 of deepTools (Ramirez, Ryan et al, 2016). All replicates showed a Pearson correlation coefficient of greater than 0.95 for transcriptionally active genes."

We now state explicitly in the legends for Figure 3D, 4B and 4D that the data are the average of two independent experiments. Also, we now include a new Figure EV2, in which the data from each experiment are plotted, together with the associated mean values and confidence intervals (described in Materials and Methods on lines 722-726).

9. Figure 3D - The level of Ser5P appears to be lower in the SPT5-3A strain. Can the authors comment on this? It's possible that no strong conclusion can be made due to the absence of spike-in normalization; however, given that there are effects seen later in the MNase-seq and H3K4me3 ChIP-seq experiments, it is of great interest to know if there is any correlation of effects on transcription with these later results. For example, since the H3K4me3 effects were greatest for the most highly transcribed quartile, what happens with the Ser5P profile if it is divided into quartiles? Given that the Spt5N-3A mutant fails to bind histones, it is reasonable to assume that its primary defect in vivo is on chromatin structure, but it cannot be ruled out that its primary effect is on transcription, with changes in chromatin structure as a consequence.

We are grateful to the reviewer for discussing this point, as it made us realise that unfortunately we had made an error in our normalisation of the data in Figure 3D. We apologise for this error and we now present the corrected version, which shows that SPT5-3A and SPT5 are actually very similar (see new version of Figure 3D). Inactivation of Spt5-AID without any expression of exogenous Spt5 (whether wt or 3A) leads to a relative accumulation of Rpb1 at the 5' end of genes, reflecting the predicted elongation defect. We cannot make absolute comparisons of signal levels between strains in the absence of spike-in normalisation.

10. In Methods, the authors explain that their data analysis is performed on 821 transcriptionally active genes. This aspect of the analysis should be presented in results, as most omics analyses would include a larger number of genes. They should also make this clear in the figure legends.

We now make this clear in the Results on lines 288-289, the legend to Figure 3 on lines 1013-1014 and the legend to Figure 4 on lines 1030-1031.

11. For all ChIP-seq and MNase-seq experiments, Western analysis should be presented to test whether the proteins being assayed have normal levels. This is clearly relevant for the Ser5P signal after Spt5 depletion, given the two recent studies that showed that Spt5 depletion causes depletion of RNAPII (Aoi et al. and Hu et al., Mol. Cell). Also, for the MNase-seq experiments, it's conceivable that there is a reduced level of histones in the spt5-AID samples where the signal is reduced. The Western in 3B suggests that there is a normal level of H3K4me3, but that Western did not include total levels of histone H3, nor did it include either a good loading control or statistical analysis.

We now present new data in Appendix Figure S1A to show that depletion of Spt5-AID does not lead to destabilisation of Rpb1 or histones. These findings are discussed on lines 277-279 and we cite the recent work from Aoi et al and Hu et al.

Referee #2:

In this manuscript, Ervin, Serra-Caradona et al. show that the highly conserved and essential Spt5 transcription elongation factor contains a histone-binding motif that is required for cell viability. The authors further show that this domain is required to prevent the loss of nucleosomal histones and maintenance of histone H3K4me3 in actively transcribed genes. The findings establish a role for Spt5 in histone transfer in addition to its previously described role in processive transcription. This is an excellent paper that provides new insight into the biochemical functions of a highly conserved transcription-associated protein. The new insights about potential parallels in mechanisms of histone capture and transfer during transcription involving similar roles for Spt5 and Mcm2 during transcription and replication, respectively, are very interesting. The results are mostly straightforward and support the key conclusions of the paper.

Specific comments

1. The authors conclude based on MNase-seq results that Spt5 is required for maintenance of nucleosomal histones in transcribed regions. The analysis of the MNase-seq results seems incomplete. In Figure 4A, they show an example of nucleosome loss at the YFL026W genes after Spt5-AID depletion. They show provide examples of the effect of Spt5 depletion at a transcriptionally active versus lowly transcribed or transcriptionally silent (such as the silent mating type loci) genes to test their hypothesis that Spt5 is required to maintain nucleosomes during transcription. The model is of course very reasonable, but as the data are presented, direct support for transcription coupled loss of nucleosomes is not included.

We now present additional data in a new Figure EV3, for two highly expressed genes, two low expressed genes and two inactive genes.

2. In Figure 3A-D, the authors present data on the effect of Spt5 depletion and the inability of GAL-SPT5-3A histone binding mutant to rescue nucleosome loss observed after Spt5 depletion. They should also include data for the expected rescue of Spt5 depletion by wild-type GAL-SPT5 (as in Figure 2E). Otherwise, it's difficult to conclude that the observed loss of nucleosomes and H3K4me3 are caused by SPT5-3A loss of histone binding (as opposed to GAL overexpression).

Perhaps there has been some confusion here, since Figure 3A-D already contained the requested data for rescue of Spt5 depletion by wild type GAL-SPT5 (this is the spt5-AID GAL-SPT5 sample in Figure 3A-D).

Minor
1. It would be preferable to track histone loss using a tag exchange strategy, but I am persuaded with the authors use of H3K4me3 as a proxy for old histones.

We take the referee's point but are glad to see that she / he was persuaded by our choice of H3K4me3 as a proxy for old histones.

2. Have the authors tested whether the non-essential SPT4 affects nucleosome maintenance. Seems unlikely in light of the essential function of the histone binding of Spt5.

We didn't test Spt4 directly, as our project focussed on the histone-binding motif that we identified in the amino-terminal tail of Spt5. As discussed in the Introduction on lines 145-158, we previously found that Spt5 (but not Spt4) could form histone-dependent complexes with FACT, analogous to those formed by the MCM2 amino terminal tail. This was our original reason for focussing on Spt5 (rather than Spt4). Also, Spt5 histone binding activity is essential, whereas Spt4 is not an essential gene.

Referee #3:
Evrin et al. provide extensive evidence indicating that Spt5 has a histone-binding domain that is selective for H3-H4, that this domain is essential for viability in yeast, and that it is also required to maintain normal nucleosome occupancy and modification states downstream of transcription start sites. They show that histone binding is not an essential part of the elongation function of Spt5, as active Pol II occupancy within transcription units remained relatively normal after cells were forced to rely on the mutant form lacking the binding domain. This results in a very interesting suggestion that Spt5 is a key part of the histone chaperoning chain that also involves Spt6 and FACT during transcription, which is in line with the recent structures from the Cramer lab. The data are of high quality and answer some long-standing questions while also adding new pieces to the puzzle regarding how polymerases manage to progress through chromatin and then restore its properties. This story will be of broad interest to a wide audience. Some additional analysis and discussion could enhance the manuscript, as described below.

1) The correlation between the effects of the spt5N-3A mutation and transcription frequency on H3K4me3 depopulation in Fig 4F was surprisingly small, but this may have to do with the way the data are presented. Instead of plotting occupancy in the mutant against the WT, perhaps the fold change in mutant/WT plotted against transcription would be more striking?

We thank the referee for this helpful suggestion, and we have now plotted the data as suggested, in the new version of Figure 4F. We hope that the referee will agree that the new version is indeed more striking.

2) I was unable to find any discussion regarding how the residues were chosen for mutation in the spt5N-3A mutation. The acidic region with embedded hydrophobic residues is reminiscent of the interaction of FACT with H2A-H2B and the residues mutated look like they might be based on the same principles. However, interactions with H3-H4 are expected to be distinct as H3-H4 makes more extensive and stronger contact with DNA than H2A-H2B does. It would therefore be interesting to others in the field to know what else the authors tried or how they settled on the residues chosen to see if general principles can be extracted.

We were guided by our past work with the amino-terminal tail of the replication factor Mcm2. As discussed in the Introduction, we previously defined a histone-binding motif in Mcm2N (Foltman et al, 2013), which comprises an acidic region with a pair of aromatic residues that are conserved in all eukaryotes. We showed that mutation of these two very highly conserved residues abolishes histone-binding activity and subsequent work showed that this motif binds to H3-H4 (dependent upon the two conserved aromatic residues; Huang et al, 2015; Richet et al, 2015 – both cited in the Introduction).

Also as discussed in the Introduction, we found that Mcm2N could bind in yeast cell extracts to histones released from chromatin, together with one additional factor, namely FACT. Furthermore, we purified such complexes based on pull-down of FACT after release of histones from chromatin, and found by mass spectrometry that FACT was part of two analogous but mutually exclusive complexes that were both histone-dependent: one was FACT-histones-Mcm2 and the other was FACT-histones-Spt5 (Foltman et al, 2013). This suggested to us that Spt5 might be a new histone-binding factor, with an overlapping histone-binding site to Mcm2N.

Like Mcm2N, we noticed that Spt5 has an acidic amino terminal tail (Spt5N) with a small number of conserved residues that are invariant in ~ all eukaryotes. These are the three residues that we mutated to make the SPT5-3A allele. Further analysis indicated that the aromatic residue F180 is the most important of these three (our unpublished data), again reminiscent of the histone-H3-H4-binding motif of Mcm2N (small acidic motif with key aromatic residues that are invariant in diverse eukaryotes).

We have now altered the legend to Figure 1 to explain more explicitly that the three residues mutated in Spt5N-3A are the three residues within Spt5N that are conserved in ~ all eukaryotes.

3) In line 143, the Endnote citation was not converted to a reference as intended.

Thank you for pointing this out - we have now fixed it.

<u>Referee #4:</u>

This study identified a histone binding motif at the Spt5 proteins and found that this histone binding ability contributes to chromatin integrity during gene transcription. They found that Spt5-N terminus can bind H3-H4 in vitro and this motif is required for cell's viability. They also found that mutations at this binding site led to a nucleosomal histone loss during gene transcription. Therefore, they proposed a new role of Spt5 in capture and re-deposition histones during gene transcription. In general, this study is well controlled and provides novel information regarding molecular mechanisms of preserving chromatin during gene transcription. To further clarify this mechanism, it would be better to perform the following tests:

1, As the authors mentioned, Spt5N and FACT can bind simultaneously to histones released from chromatin. IP results also showed that Spt5N-3A mutant proteins lost the interactions with histones, as well as with FACT proteins. Is this suggested that the Spt5-FACT interaction is bridged by H3-H4? Whether the FACT bound histones is reduced in spt5-3A mutant cells?

The data in Figure 1B-E show that Spt5N binds directly to histones H3-H4 in the absence of other factors, dependent upon the residues mutated in Spt5-3A. The data in Figure 1F-G show that Spt5N does not interact directly with FACT in yeast cell extracts (Spt5N does not co-purify with FACT from yeast extracts, when

histones are not released by DNase treatment). In contrast, both Spt5N and FACT can bind simultaneously to the same chromatin-derived histone complexes in yeast extracts treated with DNAse, as seen previously for Mcm2N and FACT (Foltman et al, 2013). We agree with the referee that Spt5N is bridged by histones to FACT, since Spt5N-histone-FACT complexes are dependent upon the histone-binding motif of Spt5 (and Spt5N and FACT do not interact directly).

Many studies have shown previously that FACT binds histones directly, without requiring Spt5 to do so. Naturally, therefore, the ability of FACT to bind to histones in yeast extracts is not affected by degradation of Spt5-AID or expression of Spt5-3A (Figure for referees only). These represent a mixture of complexes such as FACT-histones and FACT-histones-Mcm2 (Foltman et al, 2013).

2. Since FACT is also involved in preventing nucleosomal histone H3-H4 loss during gene transcription, it would be better to check the status of FACT association at gene body regions.

We take the referee's point and it would have been interesting to monitor FACT, but unfortunately we did not have time for additional ChIP-Seq experiments within the timeframe of our revision.

Thank you for submitting your revised manuscript to The EMBO Journal. We sent it back to referee 1, who had raised several concerns during the initial review, and I am happy to inform you that this reviewer has already come back and is fully satisfied with the revisions of the study. We shall therefore be ready to proceed with formal acceptance and publication once a few remaining editorial points listed below have been addressed.

I am therefore returning the manuscript to you for a second round of revision, solely to allow you to incorporate the requested editorial modifications, and upload all modified files. Once we will have received the re-revised files, we should be ready to swiftly proceed with acceptance and publication of the manuscript.

REFEREE REPORTS

Referee #1:

The authors have done a good job of addressing the comments. I have no further concerns or suggestions.

2nd Authors' Response to Reviewers — 22nd Dec 2021

The authors have made all requested editorial changes.

ACCEPTED — 6th Jan 2022

Thank you for submitting your final revised manuscript for our consideration. I am pleased to inform you that we have now accepted it for publication in The EMBO Journal.

EMBO PRESS

YOU MUST COMPLETE ALL CELLS WITH A PINK BACKGROUND

PLEASE NOTE THAT THIS CHECKLIST WILL BE PUBLISHED ALONGSIDE YOUR PAPER

Corresponding Author Name: Dr. Karim Labib

Journal Submitted to: THE EMBO JOURNAL

Manuscript Number: EMBOJ-2021-109783

USEFUL LINKS FOR COMPLETING THIS FORM

http://www.antibodypedia.com
http://1degreebio.org
http://www.equator-network.org/reporting-guidelines/improving-bioscience-research-repor
http://grants.nih.gov/grants/olaw/olaw.htm
http://www.mrc.ac.uk/Ourresearch/Ethicsresearchguidance/Useofanimals/index.htm
http://ClinicalTrials.gov
http://www.consort-statement.org
http://www.consort-statement.org/checklists/view/32-consort/66-title
http://www.equator-network.org/reporting-guidelines/reporting-recommendations-for-tumo
http://datadryad.org
http://figshare.com
http://www.ncbi.nlm.nih.gov/gap
http://www.ebi.ac.uk/ega
http://biomodels.net/
http://biomodels.net/miriam/
http://jjj.biochem.sun.ac.za
https://osp.od.nih.gov/biosafety-biosecurity-and-emerging-biotechnology/
http://www.selectagents.gov/

Reporting Checklist For Life Sciences Articles (Rev. June 2017)

This checklist is used to ensure good reporting standards and to improve the reproducibility of published results. These guidelines are consistent with the Principles and Guidelines for Reporting Preclinical Research issued by the NIH in 2014. Please follow the journal's authorship guidelines in preparing your manuscript.

A- Figures
1. Data
The data shown in figures should satisfy the following conditions:

- the data were obtained and processed according to the field's best practice and are presented to reflect the results of the experiments in an accurate and unbiased manner.
- figure panels include only data points, measurements or observations that can be compared to each other in a scientifically meaningful way.
- graphs include clearly labeled error bars for independent experiments and sample sizes. Unless justified, error bars should not be shown for technical replicates.
- if n< 5, the individual data points from each experiment should be plotted and any statistical test employed should be justified
- Source Data should be included to report the data underlying graphs. Please follow the guidelines set out in the author ship guidelines on Data Presentation.

2. Captions

Each figure caption should contain the following information, for each panel where they are relevant:

- a specification of the experimental system investigated (eg cell line, species name).
- the assay(s) and method(s) used to carry out the reported observations and measurements
- an explicit mention of the biological and chemical entity(ies) that are being measured.
- an explicit mention of the biological and chemical entity(ies) that are altered/varied/perturbed in a controlled manner.

- the exact sample size (n) for each experimental group/condition, given as a number, not a range;
- a description of the sample collection allowing the reader to understand whether the samples represent technical or biological replicates (including how many animals, litters, cultures, etc.).
- a statement of how many times the experiment shown was independently replicated in the laboratory.
- definitions of statistical methods and measures:
  - common tests, such as t-test (please specify whether paired vs. unpaired), simple χ2 tests, Wilcoxon and Mann-Whitney tests, can be unambiguously identified by name only, but more complex techniques should be described in the methods section;
  - are tests one-sided or two-sided?
  - are there adjustments for multiple comparisons?
  - exact statistical test results, e.g., P values = x but not P values < x;
  - definition of 'center values' as median or average;
  - definition of error bars as s.d. or s.e.m.

Any descriptions too long for the figure legend should be included in the methods section and/or with the source data.

In the pink boxes below, please ensure that the answers to the following questions are reported in the manuscript itself. Every question should be answered. If the question is not relevant to your research, please write NA (non applicable). We encourage you to include a specific subsection in the methods section for statistics, reagents, animal models and human subjects.

B- Statistics and general methods

Please fill out these boxes (Do not worry if you cannot see all your text once you press return)

1.a. How was the sample size chosen to ensure adequate power to detect a pre-specified effect size?	NA
1.b. For animal studies, include a statement about sample size estimate even if no statistical methods were used.	NA
2. Describe inclusion/exclusion criteria if samples or animals were excluded from the analysis. Were the criteria pre-established?	NA
3. Were any steps taken to minimize the effects of subjective bias when allocating animals/samples to treatment (e.g. randomization procedure)? If yes, please describe.	NA
For animal studies, include a statement about randomization even if no randomization was used.	NA
4.a. Were any steps taken to minimize the effects of subjective bias during group allocation or/and when assessing results (e.g. blinding of the investigator)? If yes please describe.	NA
4.b. For animal studies, include a statement about blinding even if no blinding was done	NA
5. For every figure, are statistical tests justified as appropriate?	YES
Do the data meet the assumptions of the tests (e.g., normal distribution)? Describe any methods used to assess it.	NA
Is there an estimate of variation within each group of data?	NA

Is the variance similar between the groups that are being statistically compared?	NA

C- Reagents

6. To show that antibodies were profiled for use in the system under study (assay and species), provide a citation, catalog number and/or clone number, supplementary information or reference to an antibody validation profile. e.g., Antibodypedia (see link list at top right), 1DegreeBio (see link list at top right).	DETAILS ARE PROVIDED IN TABLE EV1
7. Identify the source of cell lines and report if they were recently authenticated (e.g., by STR profiling) and tested for mycoplasma contamination.	NA

* for all hyperlinks, please see the table at the top right of the document

D- Animal Models

8. Report species, strain, gender, age of animals and genetic modification status where applicable. Please detail housing and husbandry conditions and the source of animals.	NA
9. For experiments involving live vertebrates, include a statement of compliance with ethical regulations and identify the committee(s) approving the experiments.	NA
10. We recommend consulting the ARRIVE guidelines (see link list at top right) (PLoS Biol. 8(6), e1000412, 2010) to ensure that other relevant aspects of animal studies are adequately reported. See author guidelines, under 'Reporting Guidelines'. See also: NIH (see link list at top right) and MRC (see link list at top right) recommendations. Please confirm compliance.	NA

E- Human Subjects

11. Identify the committee(s) approving the study protocol.	NA
12. Include a statement confirming that informed consent was obtained from all subjects and that the experiments conformed to the principles set out in the WMA Declaration of Helsinki and the Department of Health and Human Services Belmont Report.	NA
13. For publication of patient photos, include a statement confirming that consent to publish was obtained.	NA
14. Report any restrictions on the availability (and/or on the use) of human data or samples.	NA
15. Report the clinical trial registration number (at ClinicalTrials.gov or equivalent), where applicable.	NA
16. For phase II and III randomized controlled trials, please refer to the CONSORT flow diagram (see link list at top right) and submit the CONSORT checklist (see link list at top right) with your submission. See author guidelines, under 'Reporting Guidelines'. Please confirm you have submitted this list.	NA
17. For tumor marker prognostic studies, we recommend that you follow the REMARK reporting guidelines (see link list at top right). See author guidelines, under 'Reporting Guidelines'. Please confirm you have followed these guidelines.	NA

F- Data Accessibility

18: Provide a "Data Availability" section at the end of the Materials & Methods, listing the accession codes for data generated in this study and deposited in a public database (e.g. RNA-Seq data: Gene Expression Omnibus GSE39462, Proteomics data: PRIDE PXD000208 etc.) Please refer to our author guidelines for 'Data Deposition'.<br><br>Data deposition in a public repository is mandatory for:<br>a. Protein, DNA and RNA sequences<br>b. Macromolecular structures<br>c. Crystallographic data for small molecules<br>d. Functional genomics data<br>e. Proteomics and molecular interactions	DONE
19. Deposition is strongly recommended for any datasets that are central and integral to the study; please consider the journal's data policy. If no structured public repository exists for a given data type, we encourage the provision of datasets in the manuscript as a Supplementary Document (see author guidelines under 'Expanded View' or in unstructured repositories such as Dryad (see link list at top right) or Figshare (see link list at top right).	DONE
20. Access to human clinical and genomic datasets should be provided with as few restrictions as possible while respecting ethical obligations to the patients and relevant medical and legal issues. If practically possible and compatible with the individual consent agreement used in the study, such data should be deposited in one of the major public access-controlled repositories such as dbGAP (see link list at top right) or EGA (see link list at top right).	NA
21. Computational models that are central and integral to a study should be shared without restrictions and provided in a machine-readable form. The relevant accession numbers or links should be provided. When possible, standardized format (SBML, CellML) should be used instead of scripts (e.g. MATLAB). Authors are strongly encouraged to follow the MIRIAM guidelines (see link list at top right) and deposit their model in a public database such as Biomodels (see link list at top right) or JWS Online (see link list at top right). If computer source code is provided with the paper, it should be deposited in a public repository or included in supplementary information.	NA

G- Dual use research of concern

22. Could your study fall under dual use research restrictions? Please check biosecurity documents (see link list at top right) and list of select agents and toxins (APHIS/CDC) (see link list at top right). According to our biosecurity guidelines, provide a statement only if it could.	NO
